# Reversible, Red-Shifted Photoisomerization in Protonated Azobenzenes

**DOI:** 10.1021/acs.joc.2c00661

**Published:** 2022-08-03

**Authors:** Jonas Rickhoff, Niklas B. Arndt, Marcus Böckmann, Nikos L. Doltsinis, Bart Jan Ravoo, Luuk Kortekaas

**Affiliations:** † Organisch-Chemisches Institut, Westfälische Wilhelms-Universität Münster, Corrensstraße 36, 48149 Münster, Germany; ‡ Center for Soft Nanoscience, Westfälische Wilhelms-Universität Münster, Busso-Peus-Straße 10, 48149 Münster, Germany; § Institute for Solid State Theory and Center for Multiscale Theory & Computation, Westfälische Wilhelms-Universität Münster, Wilhelm-Klemm-Str. 10, 48149 Münster, Germany; ∥ Materials Chemistry, Faculty of Science and Engineering, University of Groningen, Nijenborgh 4, 9747 AG Groningen, The Netherlands

## Abstract

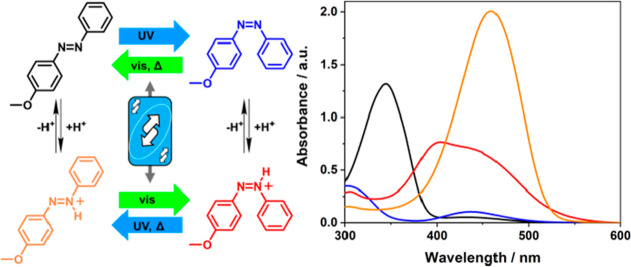

Azobenzenes are among the best-studied molecular photoswitches and play a key role in the search for red-shifted photoresponsive materials for extended applications. Currently, most approaches deal with aromatic substitution patterns to achieve visible light application, on occasion paired with protonation to yield red-shifted absorption of the azonium species. Appropriate substitution patterns are essential to stabilize the latter approach, as conventional acids are known to induce a fast *Z*- to *E*-conversion. Here, we show that steady-state protonation of the azo-bridge instead is possible in simple azobenzenes when the p*K*_a_ of the acid is low enough, yielding both the *Z*- and *E*-azonium as supported by UV–vis- and ^1^H NMR spectroscopy as well as density functional theory calculations. Moreover, the steady-state protonation of *para*-methoxyazobenzene, specifically, yields photoisomerizable azonium ions in which the direction of switching is essentially reversed, that is, visible light produces the out-of-equilibrium *Z*-azonium. Although the current conditions render the visible light photoswitch unsuitable for in vivo and material application, the demonstrated understanding of simple azobenzenes paves the way for a great range of further work on this already widely studied photoswitch.

## Introduction

**Figure 1:**
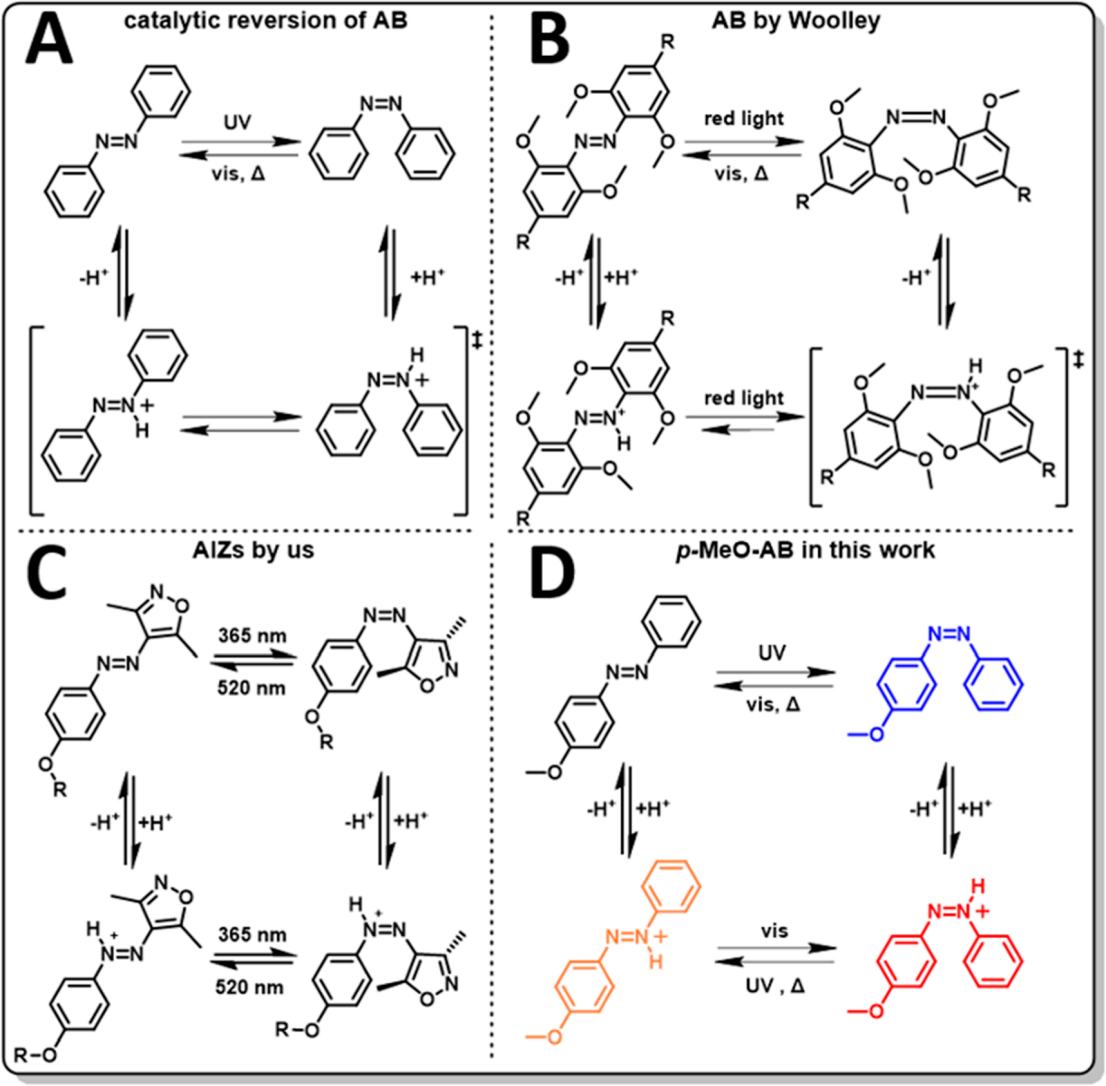
Isomerization schemes of pH-controlled photochromism in ABs and AIZs in previous works^25,28−32^ (A–C) and *p*-MeO-AB in this work (D). Instead of conventional acids such as HCl and CF_3_COOH, the steady states in C and D were achieved with CF_3_SO_3_H. ‡ = not observed.

For decades, a key interest has been taken in the material incorporation of functional molecules, that is, molecules that exhibit a change in a host of physicochemical properties in response to external stimuli.^1,2^ These materials most commonly react to light of different wavelengths,^3−5^ pH changes,^6,7^ or redox potential,^8^ due to the wide range of such building blocks available and their ease of applicability. As such, light is one of the most often used and sought-after triggers for functional materials because this noninvasive stimulus offers spatiotemporal control at low operational costs.^9^ Molecules that photoreversibly change between at least two distinguishable (meta)stable states are referred to as photoswitches, with spiropyrans,^5,10^ diarylethenes,^4^ and azobenzenes^11,12^ being among the most widely applied. Azobenzenes (ABs), especially, have proven to be outstanding building blocks for functional materials owing to their modularity, reflecting also in excellently tunable property changes.^13−16^ At their core, azobenzenes consist of two benzene units connected via an azo N=N double bond, which, upon preventing free rotation, yields two observable stereoisomers typically separated by about 48 kJ·mol^–1^.^17,18^ The first isolation of the metastable Z-isomer of plain as well as several substituted azobenzenes succeeded in 1935 by Hartley.^19^ At this time, Hartley already noted a substituent effect on relaxation rates, a key property that has been extensively utilized to ultimately yield tunable absorption properties, thermal relaxation rates, fluorescence, and polarity within this class of photochromes.^17−21^ Although azobenzenes are mostly used for their *E*–*Z*-isomerization properties, it is worth noting that through the introduction of intramolecular hydrogen bonding^22^ or chalcogen bonding,^23^ the isomerization process can be inhibited altogether. In many recent works, increased stability of both isomers is also achieved by the introduction of a heterocycle replacing one of the phenyl rings to form, for example, arylazopyrazoles (AAPs)^24,25^ and azoisoxazoles (AIZs)^26^ that exhibit superior thermal lifetimes and quantitative switching in both directions. Here, we seek to further investigate the role of pH in manipulating azobenzene photophysics. Classically, the addition of conventional acids and specific metal salts is known to destabilize the *Z*-azobenzene form at varying rates, depending on the type as well as amounts added.^19^ The thermal half-life of *Z*-AAPs has also been aptly shown to be tunable using pH adjustments by the groups of Fuchter^27^ and Walther.^25^ In recent work of our own, we reported the pH-gated photoisomerization for AIZ-derivatives, where a strong acid was added to *para*-alkoxy azoisoxazoles to unlock an isomerization pathway via the protonated *E*- and *Z*-isomers.^28^ This latest work ultimately led us to revisit and update the pH-response of azobenzenes in the current contribution (Figure 1).

The kinetics of the acid-catalysis in azobenzenes is generally believed to be of the first order, showing deviations at high catalyst loading, and the catalytic action of different acids and salts was found to have a cumulative effect.^33,34^ Protonation of either azo nitrogen of the *Z*-isomer was found to be the key to decreasing the double bond character and enabling fast isomerization with immediate deprotonation due to increased acidity of the *E*-isomer (Figure 1A).^19,33,34^ The use of electron-donating substituents in the para position to the azo-functionality also increases the reversion rate owing to the resulting increase in the basicity of the azo-moiety.^35^

Although the catalytic activity of protons in the reversion process of azobenzenes has, thus, been well-established, some amino-azo dyes, for example, pH indicators and optical sensors, are known to form stable protonated states in aqueous media with red-shifted absorptions.^36−38^ Light responsiveness in such red-shifted azobenzenes is of great interest for applications in biological systems due to the hazard of the conventional UV-triggered photoswitching to living organisms, and the improved tissue penetration of red light.^3,39^ Most notably, Woolley et al. have succeeded in developing such red-shifted photoresponse in tetramethoxy-azobenzenes, enabling protonation at a pH of 4.8 owing to both an increased electronegativity of the azo bond and multiple H-bonding opportunities for the azonium ion (Figure 1B). These protonated azonium ions show a significant red shift in absorbance with a λ_max_ at 560 nm, which reversibly decreases upon visible light irradiation. These findings line up well with the red shift that Woolley reported for azonium ions, constituting a λ_max_ > 600 nm.^35^ Although the thermal relaxation of the *Z*-azonium to the corresponding *E*-form takes place, their density functional theory (DFT) calculations indicate that deprotonation of the former should also be possible because of the poorer H-bonding in the *Z*-state, yielding the unprotonated form upon isomerization. Ultimately, they show excellent cyclability without photobleaching,^40^ and, in further work, demonstrate that by varying the substituents on the benzene rings, the absorption wavelength and the p*K*_a_ value of the protonated *E*-isomer can be tuned.^41^

In this work, we investigated the photophysical behavior of plain azobenzene as well as its derivatives *p*-O_2_N-AB and *p*-MeO-AB under the influence of conventional and stronger acids. As previously reported for ABs,^34,42^ the catalytic reversion with protons holds for all three ABs when weak enough acids are employed. Additionally, and similar to our results for AIZs (Figure 1C),^28^ strong acids such as CF_3_SO_3_H (trifluoromethane sulfonic acid, TfOH) produce the azonium forms of AB, *p*-O_2_N-AB and *p*-MeO-AB, with particular retention of photophysics in the methoxy derivative (Figure 1D). Furthermore, the newly accessible protonated *E*- and *Z*-MeO-ABH^+^ ions show a reverse response with respect to the unprotonated forms, exhibiting vis-induced *E*- to *Z*- isomerization and UV-induced and thermal *Z*- to *E*-isomerization, as supported by UV–vis and NMR spectroscopy as well as DFT calculations. In the current pursuit of easily accessible photoswitches that may be addressed with longer wavelengths, we believe that this contribution will greatly benefit further development of their material applications.

## Results

### Catalytic *Z*- to *E*-Reversion with Conventional Acids

**Figure 2:**
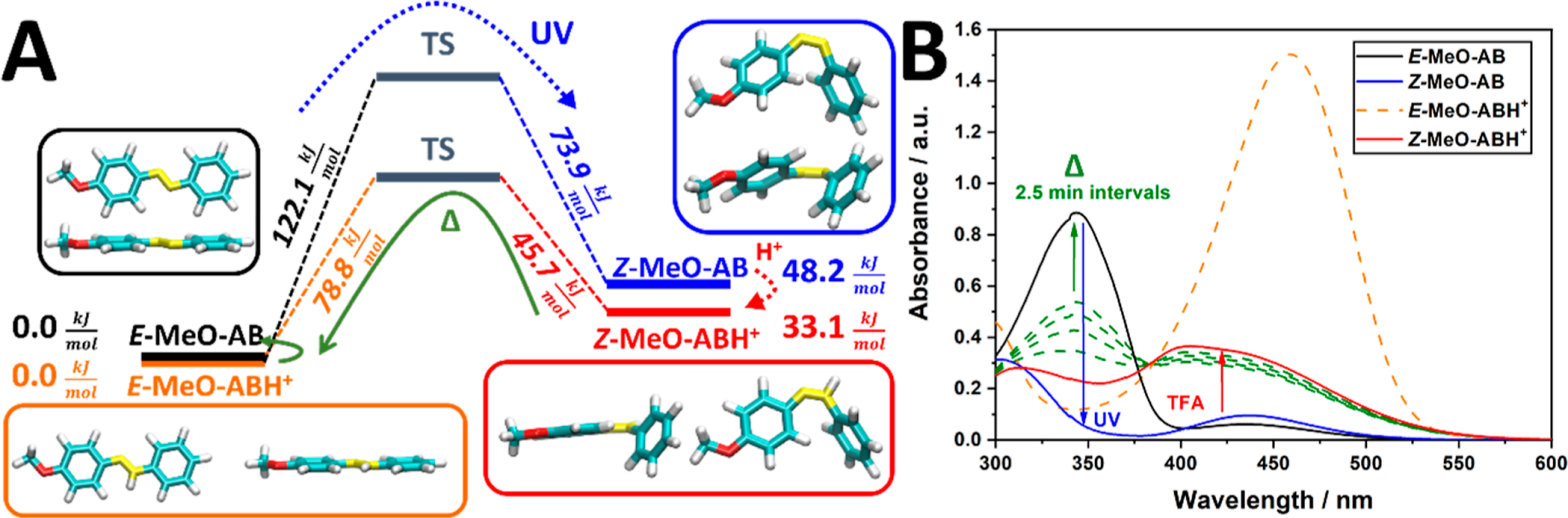
(A) Relative Gibbs free energies of *E*- and *Z*-isomers of MeO-AB, and MeO-ABH^+^ and their respective transition states^45^ (see also Figure S3. For AB and O_2_N-AB see Figure S4); (B) UV–vis-spectra of *E*-MeO-AB (41 μM in acetonitrile) before (black) and after irradiation at 365 nm to form *Z*-MeO-AB (blue). Addition of excess TFA leads to the formation of *Z*-MeO-ABH^+^ (red), with thermal reversion back to *E*-MeO-AB (green, dashed) over the transient *E*-MeO-ABH^+^ form; for reference, the absorbance of *E*-MeO-ABH^+^, acquired by direct addition of excess CF_3_SO_3_H (TfOH) to *E*-MeO-AB (see “Steady State Formation of Azonium Ions” below) has been inserted (orange dotted).

To set the stage, we will first consider the *Z*- to *E*-reversion of MeO-AB through general acid catalysis with conventional acids. Although the thermal equilibrium of MeO-AB in acetonitrile contains 38% of the *Z*-isomer (Figure S1), and 4% of the *E*-isomer remains at the PSS_365nm_ (Figure S2), for the purpose of readability, we consider the major species in our nomenclature in case of *E*-MeO-AB. As shown in previous studies, protonation of the azo bridge with, for example, trifluoroacetic acid (TFA) or hydrochloric acid (HCl) drastically lowers the half-life of *Z*-azobenzene due to destabilization of the double N=N bond, resulting in a reduced isomerization barrier (see Table S1).^43,44^ We note, however, that strictly seen the protonated species should be considered to have an equilibrium separate from the unprotonated species, having disparate energies and, moreover, energy barriers for isomerization (Figure 2, Table S1).

Indeed, our DFT calculations confirm a drop in the energy barrier for the *Z*- to *E*-isomerization from 73.9 kJ·mol^–1^ for the unprotonated species to 45.7 kJ·mol^–1^ for the corresponding azonium species, through which a more rapid equilibration between *Z*-MeO-ABH^+^ and *E*-MeO-ABH^+^ would establish (Figure 2A). This equilibrium, however, is drained immediately by the deprotonation of the more acidic *E*-MeO-ABH^+^, which indeed cannot be observed by steady-state spectroscopy. The systems in this work isomerize predominantly via rotation as seen in Figures S5–S8. It is clearly visible from these energy landscapes that a change in the CNNC-dihedral angle (commonly termed “rotation”) is preferred over the CNN-bond angle (commonly termed “inversion”) change.^46^

Although *Z*-AB was found to merely undergo direct catalytic reversion with protons upon addition of TFA (see Figure 1A), *Z*-O_2_N-AB and *Z*-MeO-AB exhibit a partial absorption of the azonium form, as supported by DFT (Figures S3, S4B, and S9). However, the *Z*-azonium is only subtly observed in the case of *Z*-O_2_N-ABH^+^ due to a lower absorption coefficient (Figure S10), and the presence of *Z*-MeO-ABH^+^ is clearly observed owing to a strong increase in molar absorptivity (Figure 2B, red line). As the *Z*-MeO-ABH^+^ signal subsequently fades over time, the original absorbance of the unprotonated *E*-form is restored with an isosbestic point at 383 nm, showing that there are no second steady-state species, for example, the *E*-MeO-ABH^+^, present during the reversion process. The complete destabilization of the protonated equilibrium is additionally confirmed by ^1^H NMR spectroscopy, where the addition of 15 equiv of TFA causes all signals corresponding to the *Z*-isomer to quantitatively convert to *E*-MeO-AB (Figure S11). This indicates that the p*K*_a_ of AB in the *E*-form is, contrary to the *Z*-form, significantly lower than that of TFA and that it is, therefore, only involved as a transient species. Notably, irradiation at 365 nm does not result in appreciable recovery of the protonated *Z*-isomer, which we will further clarify below (see “pH-Gated Photochromism”).

### Steady State Formation of Azonium Ions

**Figure 3:**
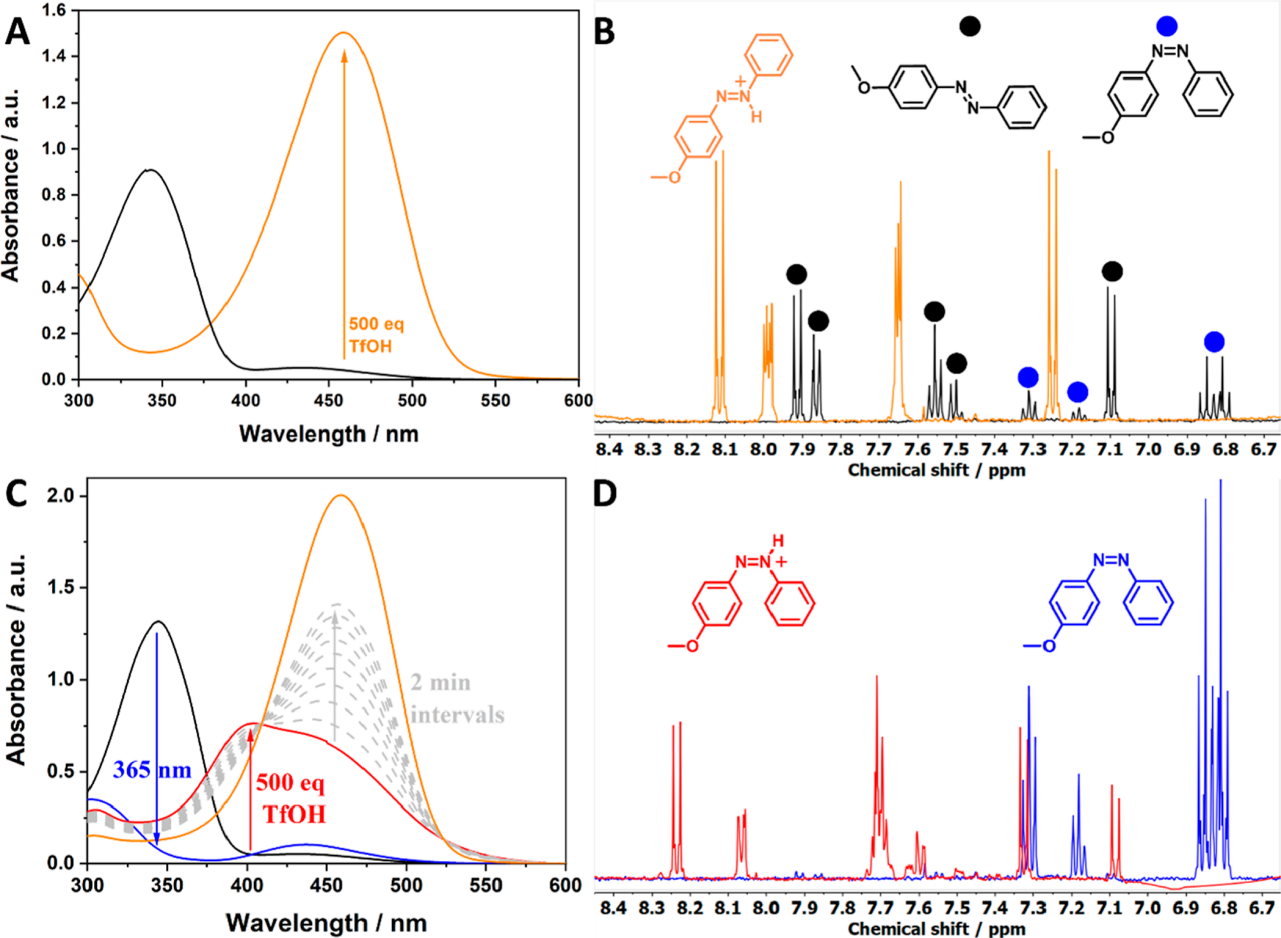
Steady-state protonation of MeO-AB with excess TfOH. (A) Absorption spectra of MeO-AB before (black) and after the addition of 500 equiv of TfOH (orange) (at 41 μM in acetonitrile); (B) NMR spectra of MeO-AB (black, mixture of isomers) and *E*-MeO-ABH^+^ (orange) (at 0.2 mM in acetonitrile-*d*_3_, 15 equiv of TfOH added to produce *E*-MeO-ABH^+^); (C) absorption spectra of ambient MeO-AB (black, mixture of isomers), MeO-AB at PSS_365nm_ (blue), and *Z*-MeO-ABH^+^ (red); thermal reversion of *Z*-MeO-ABH^+^ (grey); and the ultimate *E*-MeO-ABH^+^ absorption (orange) (at 61 μM in acetonitrile, 500 equiv TfOH added); (D) NMR spectra of *Z*-MeO-AB (blue) and *Z*-MeO-ABH^+^ (red) (at 0.2 mM in acetonitrile-*d*_3_, 15 equiv of TfOH added to produce *Z*-MeO-ABH^+^). For peak integration and coupling constants to (B,D) see Table S2.

As previously observed by Woolley et al., the absorption of AB derivatives can be significantly red-shifted when protonation of the azo bond is possible,^35^ much like that shown by our calculated UV–vis spectra of MeO-AB (Figure S12). Although we observed a clear red-shifted absorption upon addition of TFA to *Z*-MeO-AB (Figure 2), no steady-state *E*-MeO-ABH^+^ was seen in UV–vis- or NMR-spectroscopy when using conventional acids such as HCl or TFA. Upon addition of a stronger acid, CF_3_SO_3_H (TfOH), no new species of O_2_N-AB could be observed by NMR spectroscopy either (Figure S13), despite a clear red-shift in absorption through UV–vis spectroscopy when adding TfOH, specifically (Figures S15B, S16B, and S17B).^47^ Adding TfOH to AB, however, yielded a red shift in UV–vis absorption as well as a new set of signals in ^1^H NMR spectroscopy (Figures S14, S15A, and S16A). Unfortunately, no photoreversibility was observed upon *E*- to *Z*-photoisomerization of the AB-azonium. Ultimately, the electron-donating nature of the *para*-methoxy group seems to be most fitting for facilitating azonium formation, as the addition of excess TfOH to *E*-MeO-AB leads to an intense 111 nm red-shifted absorption as well as clear ^1^H NMR shifts for both azonium species (Figure 3, Table S2). This trend in reactivity can also be seen for previously reported azobenzenes, as electron-rich phenyl rings show a preferred local energy minimum for protonated nitrogen compared to more electron-poor systems (Figure S18).^35^

**Figure 4:**
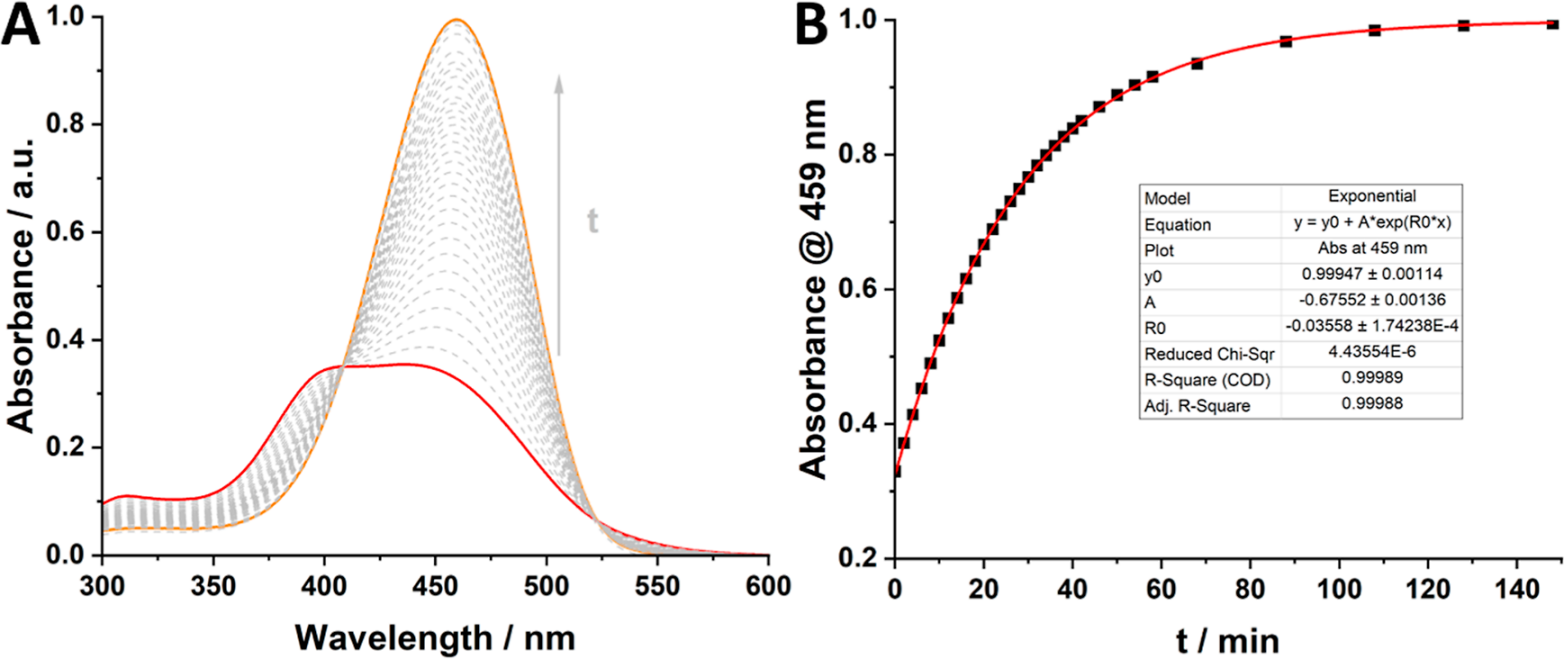
(A) UV–vis spectra of thermal *Z*- (red) to *E*-isomerization (orange) of *p*-MeO-ABH^+^ measured over time at 25 μM in acetonitrile (500 equiv TfOH was added to the PSS_365nm_ to generate *Z*-MeO-ABH^+^); (B) single wavelength absorption at 459 nm plotted against time, with an exponential fit to determine the half-life.

The observed red shifts in absorption are fully in line with DFT calculations predicting a symmetry break upon protonation of the azo bond, thus promoting the n−π*-transition (Figure S3). The key change from the catalytic reversion with protons described above is that TfOH is a strong enough acid to protonate *E*-MeO-AB, preventing the spontaneous one-way reversion to *E*-MeO-AB. Instead, a more gradual thermal reversion toward the apparently more stable *E*-MeO-ABH^+^ is observed (Figure 4), in line with the calculated order in energy.

The change in energy barriers in this new equilibrium results in a thermal half-life of the *Z*-azonium of only 19 min (Figure 4), which is several orders of magnitude shorter than for the unprotonated thermal *Z*- to *E*-isomerization (30–49 h).^19^ On the other hand, the azonium species are longer-lived than those that were previously reported to undergo reversion with concurrent deprotonation.^35,41^

### pH-Gated Photochromism

**Figure 5:**
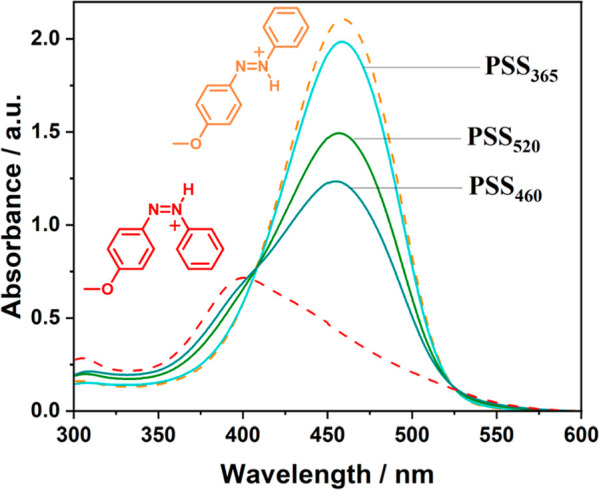
UV–vis spectra of MeO-AB azonium isomers at given photostationary states (PSSs). Spectra were recorded at 61 μM in acetonitrile. For comparison, a scaled-subtracted pure *Z*-azonium absorption was generated considering the rate of thermal reversion prior to measuring UV–vis absorption. Accordingly, PSSs are estimated to be 93% for *Z*- to *E*-isomerization (365 nm) and 36% (520 nm) and 52% (460 nm) for *E*- to *Z*-isomerization.

Most interestingly, the new absorptions of the protonated *E*- and *Z*-azobenzenes also give rise to new photophysics. AB is particularly sensitive to irradiation after protonation, degrading over time (Figures S15A, S16A, and S17A), similar to reports on photodegradation of azo-based dyes in the presence of oxygen.^48−50^ On the other hand, although irradiation of O_2_N-AB in presence of HCl or HClO_4_ yields no change in absorption (Figures S15B and S17B), irradiation in presence of TfOH photoreversibly takes the azonium forms out-of-equilibrium (Figure S16B). The changes observed for MeO-AB, however, are most intriguing, as it not only retains its photoactivity in presence of TfOH but also has its photoresponse reversed (Figure 5).

**Figure 6:**
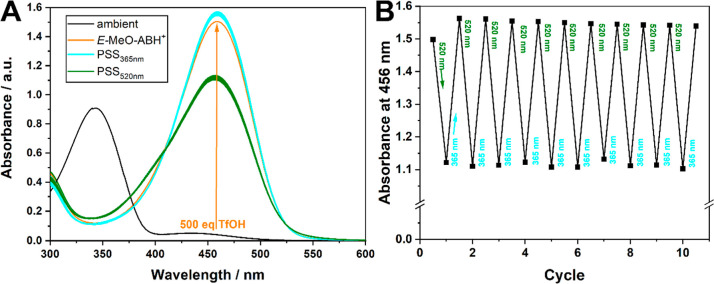
(A) UV–vis spectra of MeO-AB as ambient (black) and after addition of excess TfOH (orange), and at the subsequent PSS_365nm_ (bright blue) and PSS_520nm_ (green) (at 41 μM in acetonitrile); (B) absorbance at 456 nm over various irradiation cycles at given wavelengths.

As the order of the *Z*- and *E*-absorption bands is reversed, so is the photoisomerization, that is, 365 nm favors the *E*-form, whereas either 520 nm or 460 nm favors the *Z*-form. The higher PSS_460nm_ is at the cost of 10% fatigue over 10 cycles (Figure S19), while no significant fatigue is observed at 520 nm (Figure 6).

Interestingly, in this new photoequilibrium, it is the *E*-form that is formed as a major species (93% at 365 nm), whereas only 52% (460 nm), or to avoid fatigue 36% (520 nm), of the *Z*-form can be generated. Most important to note, however, is that the thermal equilibrium also lies strongly with the vis-responsive *E*-form, as seen by the extent of reversion observed in Figure 4. Ultimately, this means that by accessing the azonium forms, the simple AB system can now be pushed out of equilibrium at 520 or 460 nm with thermal reversion, disposing of the need for UV-irradiation altogether.

## Conclusions

In summary, we have demonstrated that the protonation of the azo-nitrogen in *p*-O_2_N-AB and *p*-MeO-AB with different acids can give rise to new thermo- and photoequilibria beyond the well-established switching behavior of azobenzenes. The key to accessing these states lies within the p*K*_a_-values of the different acids as well as the various azo-isomers, and the transition energy barriers involved. On the one hand, a catalytic reversion with protons occurs upon treating AB, *p*-O_2_N-AB, or *p*-MeO-AB with conventional acids, as shown by many studies before. On the other hand, the addition of a stronger acid, such as TfOH, causes the formation of the *E*-azonium on account of it having a higher p*K*_a_. Although AB exhibits instability in the azonium form, the O_2_N-AB and, in particular, MeO-AB azonium species are photostable upon the addition of TfOH. Ultimately, the steady-state formation of the MeO-AB azonium ions also enables access to an additional photochromic pathway with reverse photoresponse, driven out-of-equilibrium with visible light and, thereby, foregoing the need for UV-irradiation. This new understanding could greatly benefit the development of novel photochromic systems and the approach of steering azobenzenes toward a visible-light application.

## Experimental Section

### General

All chemicals and solvents were purchased from Sigma-Aldrich (Sigma-Aldrich Corp., St. Louis, Missouri, USA), Acros Organics (Fisher Scientific International, Inc., Pittsburgh, Pennsylvania, USA), and TCI (Tokyo Chemical Industry, Tokyo, Japan) and were used without further purification.

### UV–Vis Spectroscopy

UV–vis spectra were measured on a Jasco V-770 spectrophotometer (Jasco Deutschland GmbH, Pfungstadt, Germany) using high precision quartz glass cuvettes (Hellma Analytics GmbH, Müllheim, Germany). The spectra were recorded with Spectra Manager 2, Spectra Manager Version 2.14.06 (Jasco Deutschland GmbH, Pfungstadt, Germany). The samples were dissolved in the specified solvent, and the baseline was measured against the same solvent. Data analysis was done using OriginPro 2018 b b9.5.5.409 (ORIGINLAB Corporation, Northampton, USA). If not stated otherwise, the *Z*-isomer was handled in the dark or under red light.

### Irradiation and Photocyclization

Irradiation experiments were conducted using LEDs with emission wavelengths of 365 nm (UV LED Gen2 emitter, LED Engin Inc., San Jose, California, USA, radiant flux 1.2 W), 460 nm (blue LED emitter, LED Engin Inc., San Jose, California, USA, radiant flux 1.0 W), and 520 nm (LSC-G HighPower-LED, Cree Inc., Durham, North Carolina, USA, radiant flux 87 lm) at room temperature. Irradiation times of 10 s (MeO-AB) and 20 s (AB and O_2_N-AB) were used to isomerize the compounds directly inside the UV-cuvettes.

### ^1^H NMR Spectroscopy

The ^1^H NMR spectra were obtained using a DD2-600-spectrometer at 600 Hz (Agilent Technologies, Santa Clara, California, USA). Chemical shifts (δ) are reported in parts per million with respect to tetramethylsilane, referenced to residual solvent (CD_2_HCN) signals, and coupling constants are denoted in hertz. Integrations are reported, with multiplicities denoted as: s = singlet, d = doublet, t = triplet, br = broad singlet, and m = multiplet. MestReNova 14.2.0-26256 (Mestrelab Research S.L., Santiago de Compostela, Spain) was used to analyze all NMR spectra.

## Supporting Information Available

Additional UV–vis and ^1^H NMR data under the addition of different acids, calculated energies and energy barriers of all ABs, and detailed computational methods (PDF)
