# Supplementary material for: Reversible, Red-Shifted Photoisomerization in Protonated Azobenzenes
Source: J Org Chem. 2022 Aug 3;87(16):10605–12. doi: 10.1021/acs.joc.2c00661 (PMC9396658; doi:10.1021/acs.joc.2c00661)
Supplement: Supplementary file 1 — jo2c00661_si_001.pdf [file jo2c00661_si_001.pdf]

Supporting information

Reversible, red-shifted photoisomerization in protonated azobenzenes

Jonas Rickhoff,[a,b] Niklas B. Arndt,[a,b] Marcus Böckmann,[c] Nikos L. Doltsinis,[c] Bart Jan Ravoo[a,b] and Luuk Kortekaas[d,]*

[a]Organisch-Chemisches Institut, Westfälische Wilhelms-Universität Münster, Corrensstraße 36, 48149 Münster, Germany

[b]Center for Soft Nanoscience, Westfälische Wilhelms-Universität Münster, Busso-Peus-Straße 10, 48149 Münster, Germany

[c]Institute for Solid State Theory and Center for Multiscale Theory & Computation, Westfälische Wilhelms-Universität Münster, Wilhelm-Klemm-Str. 10, 48149 Münster, Germany

[d]Materials Chemistry, Faculty of Science and Engineering, University of Groningen, Nijenborgh 4, 9747 AG, Groningen, The Netherlands

[*]*E-mail: l.kortekaas@rug.nl*

Table of Contents

Supporting data S3

Supporting experimental procedures S20

General procedures S20

UV-vis spectroscopy S20

1H-NMR spectroscopy S21

DFT calculations S21

Supporting references S22

Supporting data

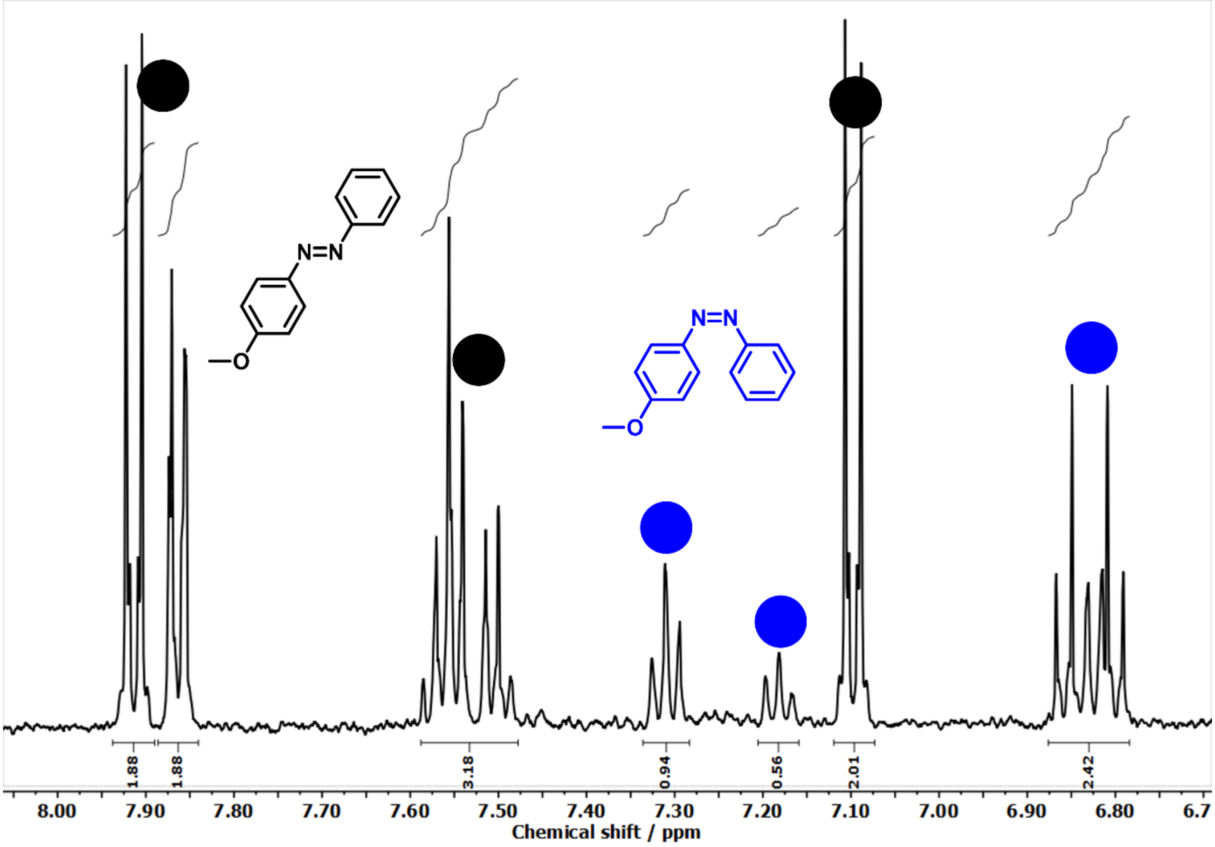

Figure S1. ^{1}H-NMR spectrum of *p*-MeO-AB (0.2 mM, 500 MHz, ACN-d_3): δ 7.94 – 7.83 (m, 4H), 7.60 – 7.47 (m, 3H), 7.33 – 7.29 (m, 1H), 7.18 (t, *J* = 7.3 Hz, 1H), 7.12 – 7.07 (m, 2H), 6.87 – 6.78 (m, 2H).

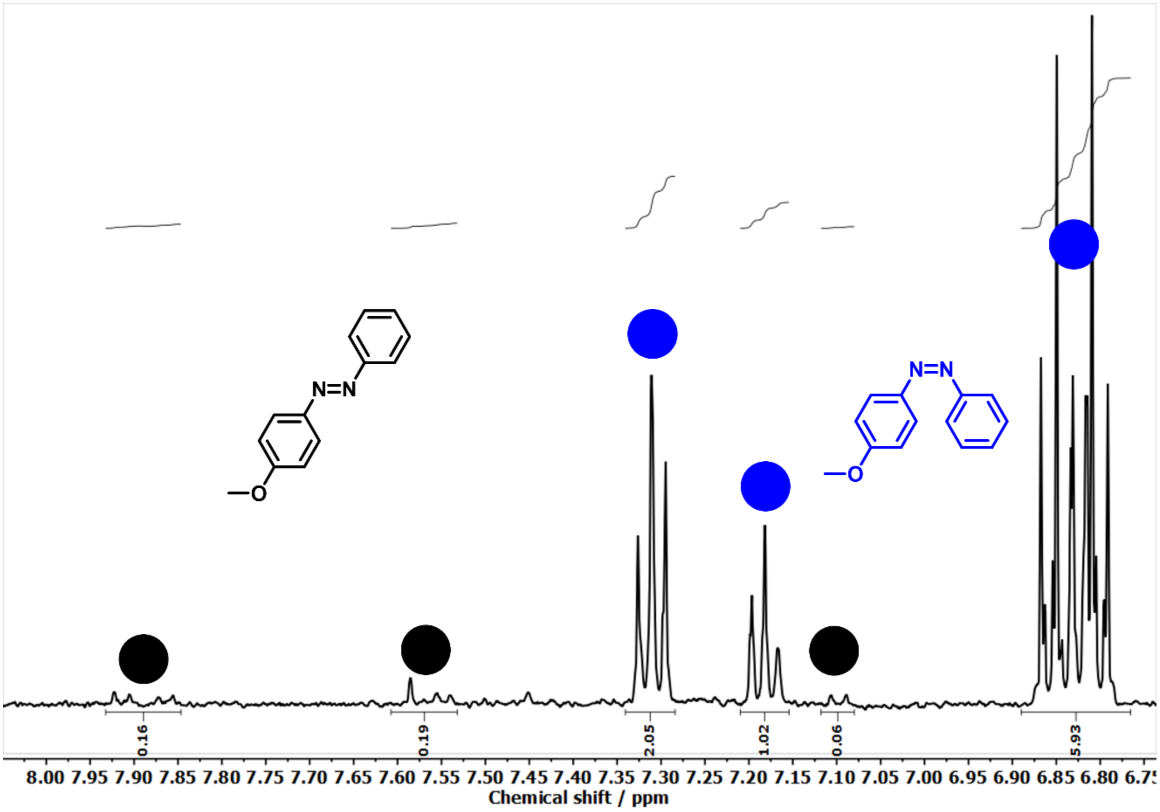

Figure S2. ^{1}H-NMR spectrum of *p*-MeO-AB at PSS_{365nm} (0.2 mM, 500 MHz, ACN-d_3): δ 7.34 – 7.28 (m, 2H), 7.18 (t, *J* = 7.3 Hz, 1H), 6.88 – 6.77 (m, 6H).

Table S1. Relative free energies of given neutral and protonated azobenzenes in kJ*mol^{-1} by DFT (PCM/GD3BJ/B3LYP/6-31G*, site ’a’ is adjacent to the phenyl ring with substituent R; ΔTS is the change w.r.t. the neutral form).

	**neutral**			**protonated**				
R=	*E*	*Z*	TS (*Z*)	*E* (b)	*E* (a)	*Z* (b)	*Z* (a)	ΔTS (*Z*)
H	0.0	44.9	132.0	0.0	-	35.2	-	-36.8
p-MeO	0.0	48.2	122.1	0.0	11.5	33.1	48.1	-43.3
p-O_2N	0.0	42.7	87.2	7.8	0.0	41.8	35.5	4.2
p-H_2N	0.0	48.9	112.9	0.0	20.8	31.3	56.3	-51.4
Tetra-*o*-MeO[1]	3.8	0.0	129.8	0.0	-	28.1	-	-46.3
Tetra-*o*-F[2]	0.0	19.3	137.7	-	-	-	-	-

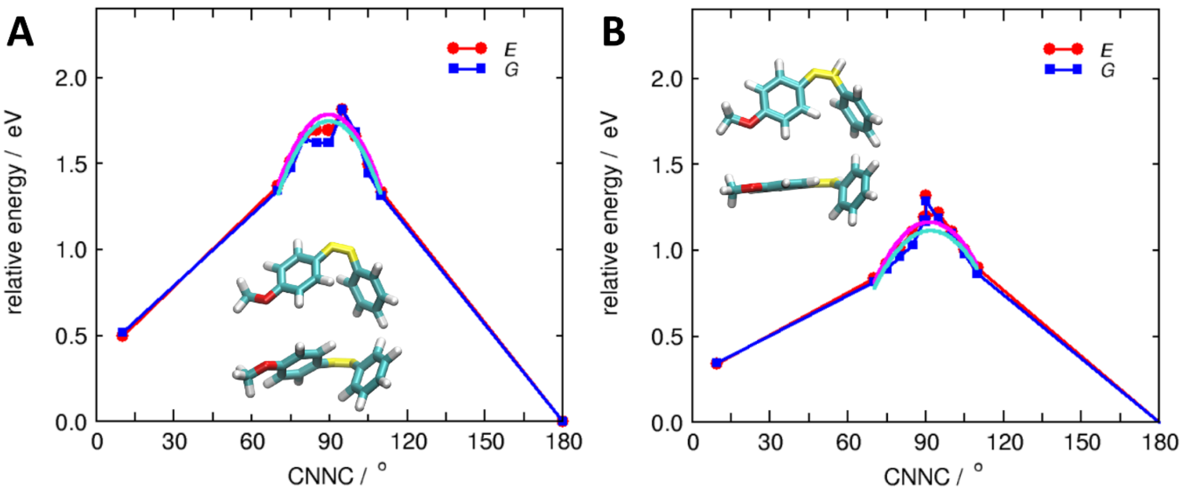

Figure S3. Relative potential energies (E) and Gibbs free energies (G) of *p*-MeO-AB (A) and *p*-MeO-ABH^+ calculated at the PCM/GD3BJ/B3LYP/6-31G* level of DFT theory; magenta and cyan lines indicate harmonic fit to obtain transition state energies and free energies respectively.

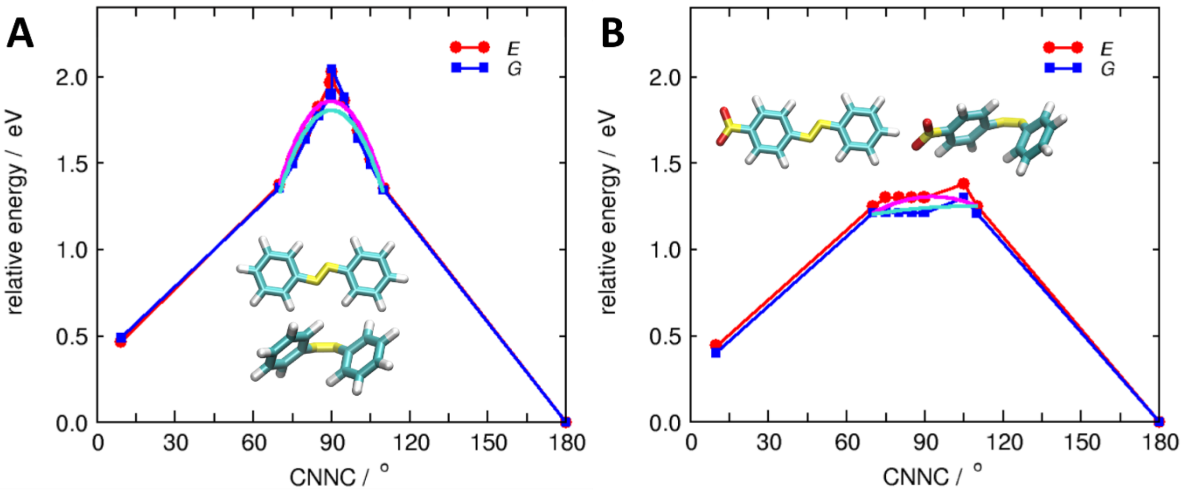

Figure S4. Relative potential energies (E) and Gibbs free energies (G) of AB (A) and *p*-O_2N-AB (B) calculated at the PCM/GD3BJ/B3LYP/6-31G* level of DFT theory; magenta and cyan lines indicate harmonic fit to obtain transition state energies and free energies respectively.

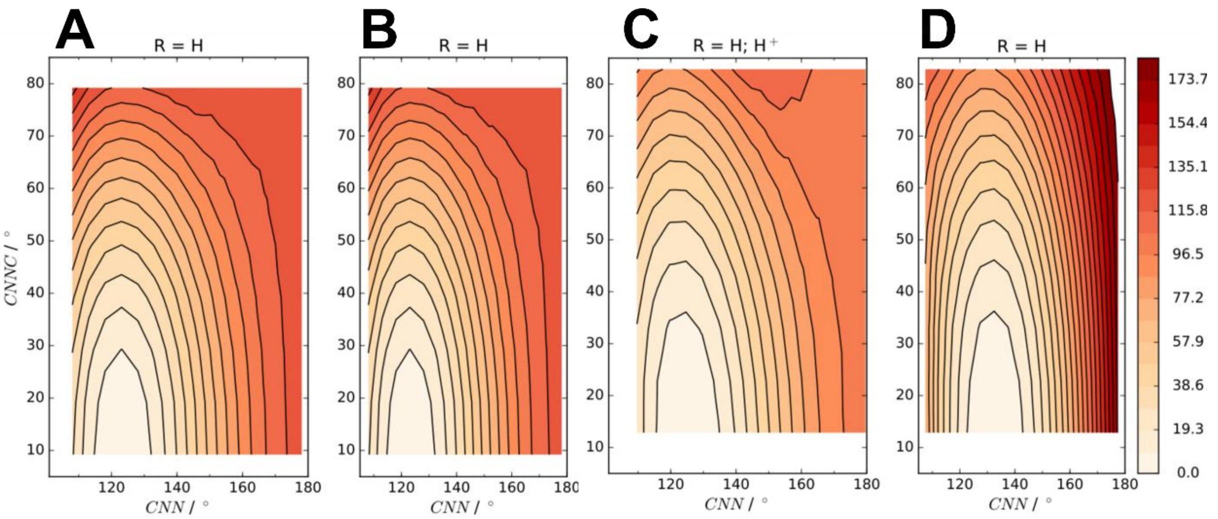

Figure S5. 2D energy surfaces for the azo bridge in AB (CNNC dihedral angle and CNN bond angle vs energy in kJ*mol^{-1}). With (A) and (B) towards unsubstituted phenyl rings, (C) towards unsubstituted phenyl ring in azonium ion (protonation site), and (D) towards unsubstituted phenyl ring in azonium ion.

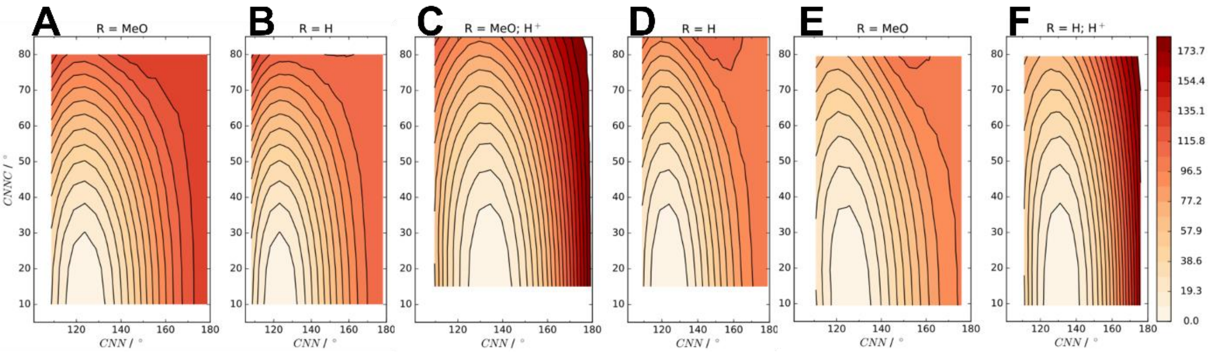

Figure S6. 2D energy surfaces for the azo bridge in MeO-AB (CNNC dihedral angle and CNN bond angle vs energy in kJ*mol^{-1}). With (A) towards the substituted phenyl ring, (B) towards the unsubstituted phenyl ring, (C) towards the substituted phenyl ring in the azonium ion (protonation site), (D) towards the unsubstituted phenyl ring in the azonium ion, (E) towards the substituted phenyl ring in the azonium ion, and (F) towards the unsubstituted phenyl ring in the azonium ion (protonation site).

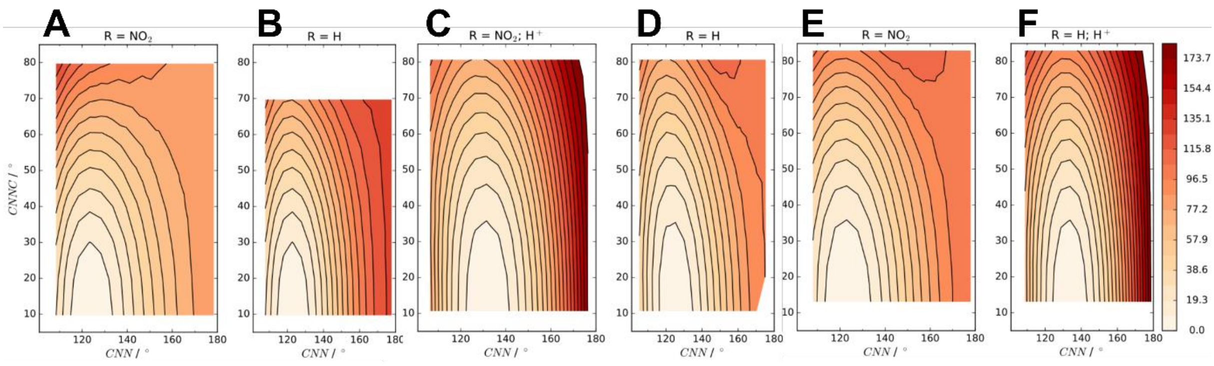

Figure S7. 2D energy surfaces for the azo bridge in O_2N-AB (CNNC dihedral angle and CNN bond angle vs energy in kJ*mol^{-1}). With (A) towards the substituted phenyl ring, (B) towards the unsubstituted phenyl ring, (C) towards the substituted phenyl ring in the azonium ion (protonation site), (D) towards the unsubstituted phenyl ring in the azonium ion, (E) towards the substituted phenyl ring in the azonium ion, and (F) towards the unsubstituted phenyl ring in the azonium ion (protonation site).

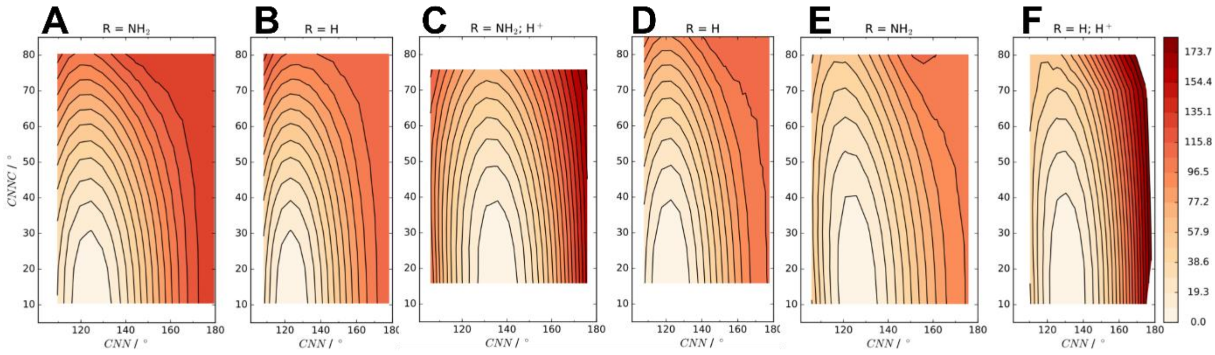

Figure S8. 2D energy surfaces for the azo bridge in H_2N-AB (CNNC dihedral angle and CNN bond angle vs energy in kJ*mol^{-1}). With (A) towards the substituted phenyl ring, (B) towards the unsubstituted phenyl ring, (C) towards the substituted phenyl ring in the azonium ion (protonation site), (D) towards the unsubstituted phenyl ring in the azonium ion, (E) towards the substituted phenyl ring in the azonium ion, and (F) towards the unsubstituted phenyl ring in the azonium ion (protonation site).

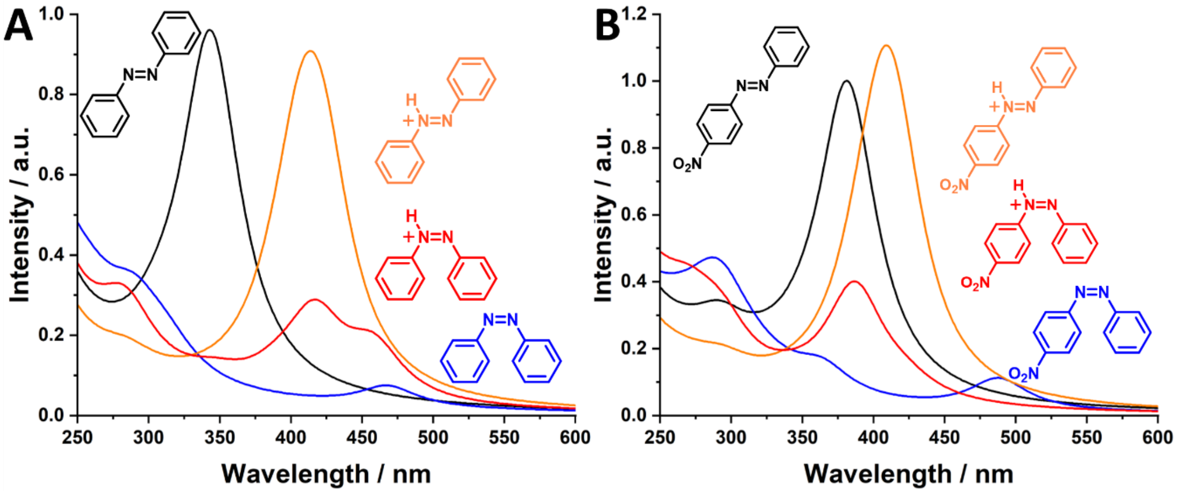

Figure S9. Calculated UV-vis spectra of AB (A) and *p*-O_2N-AB (B); *E*-isomer (black), *Z*-isomer (blue), protonated *E*-isomer (orange) and protonated *Z*-isomer (red).

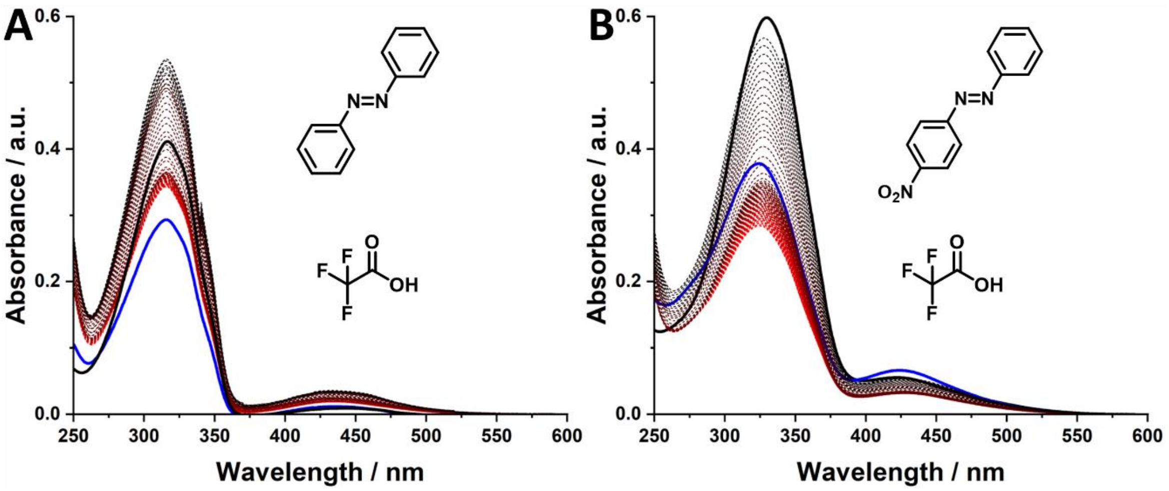

Figure S10. UV-vis spectra of AB (A) and *p*-O_2N-AB (B) at 25 µM in acetonitrile, measured in the following order: ambient (black), at PSS_{365nm} (blue), 2 min intervals after addition of 500 eq. of TFA (dotted lines from red to black).

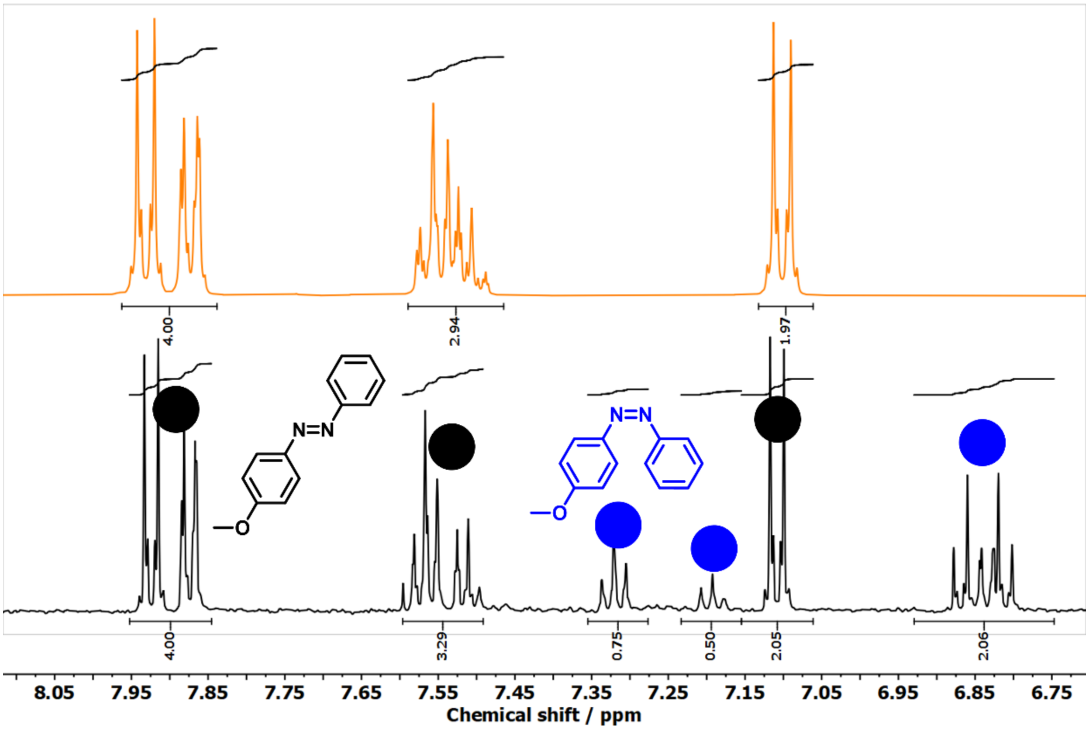

Figure S11. ^{1}H-NMR spectra of *p*-OMe-AB under ambient conditions (black line, bottom) and after addition of 15 equivalents of TFA (orange line, top) (0.2 mM, 500 MHz, ACN-d_3): δ 7.94 – 7.83 (m, 4H), 7.60 – 7.47 (m, 3H), 7.33 – 7.29 (m, 1H), 7.18 (t, *J* = 7.3 Hz, 1H), 7.12 – 7.07 (m, 2H), 6.87 – 6.78 (m, 2H).

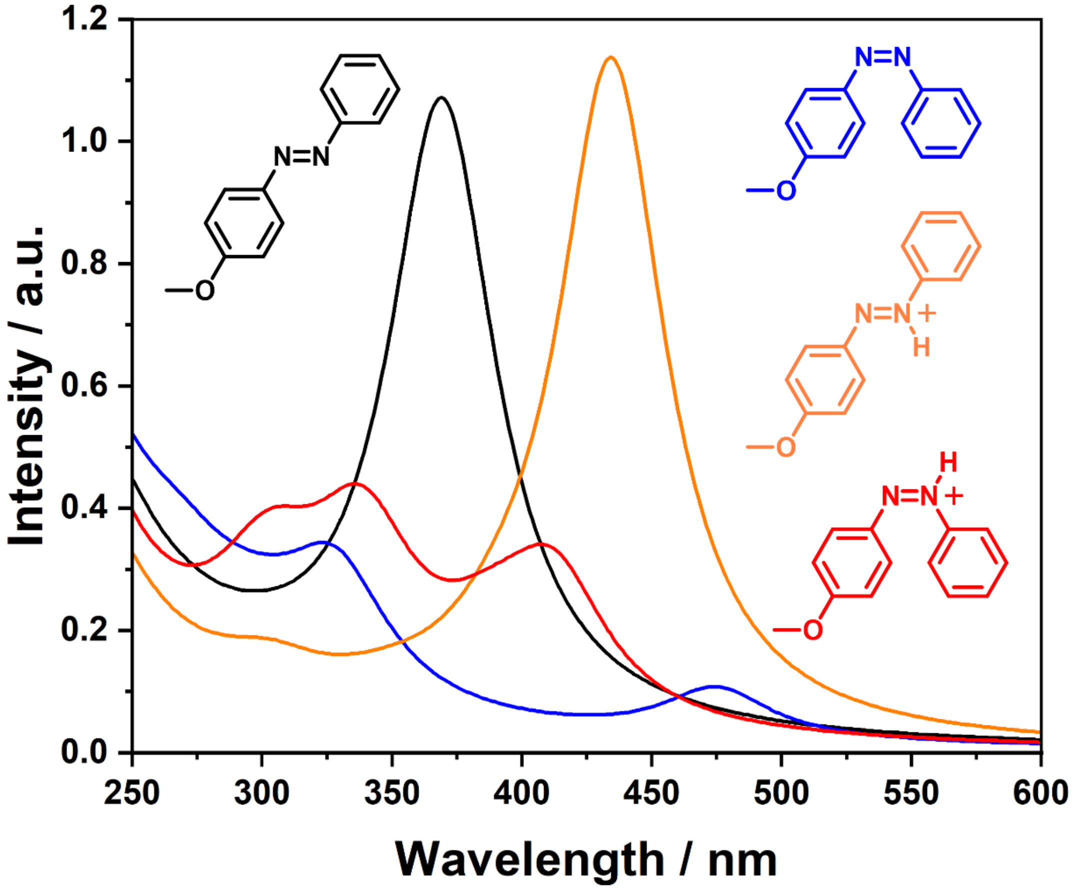

Figure S12. Calculated UV-vis spectra of p-MeO-AB; *E*-isomer (black), *Z*-isomer (blue), protonated *E*-isomer (orange) and protonated *Z*-isomer (red).

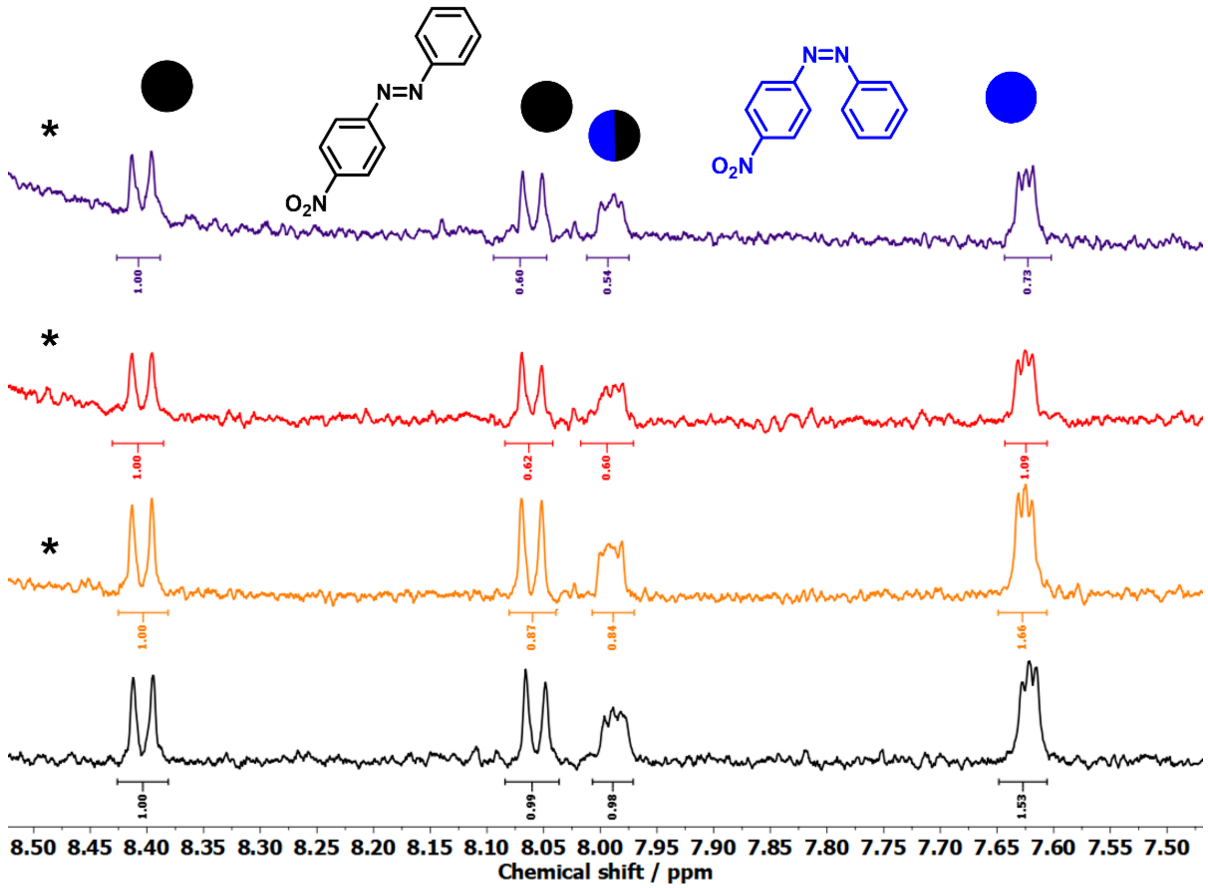

Figure S13. ^{1}H-NMR spectra of *p*-O_2N-AB under ambient conditions (black, 25 µM, 600 MHz, ACN-d_3, δ 8.40 (d, J = 8.8 Hz, 1H), 8.06 (d, J = 8.8 Hz, 1H), 7.99 (s, 2H), 7.62 (s, 2H)), after addition of 500 equivalents of TfOH (orange, 25 µM, 600 MHz, ACN-d_3, δ 8.40 (d, J = 8.7 Hz, 3H), 8.06 (d, J = 8.9 Hz, 4H), 7.98 (s, 1H), 7.62 (s, 3H)), irradiation with UV light (red, 25 µM, 600 MHz, ACN-d_3, δ 8.40 (d, J = 8.7 Hz, 2H), 8.06 (d, J = 8.9 Hz, 2H), 7.98 (s, 3H), 7.63 (s, 5H)) and irradiation with green light (purple, 25 µM, 600 MHz, ACN-d_3, δ 8.40 (d, J = 8.4 Hz, 3H), 8.06 (d, J = 8.4 Hz, 2H), 7.99 (s, 4H), 7.62 (s, 5H)). Thus, no signs of the protonated forms were observed in ^{1}H NMR spectroscopy, likely due to the less basic nature of the azo-bond in conjunction with the electron withdrawing nitro-substituent.
* broad peaks from interaction with 500 eq. TfOH.

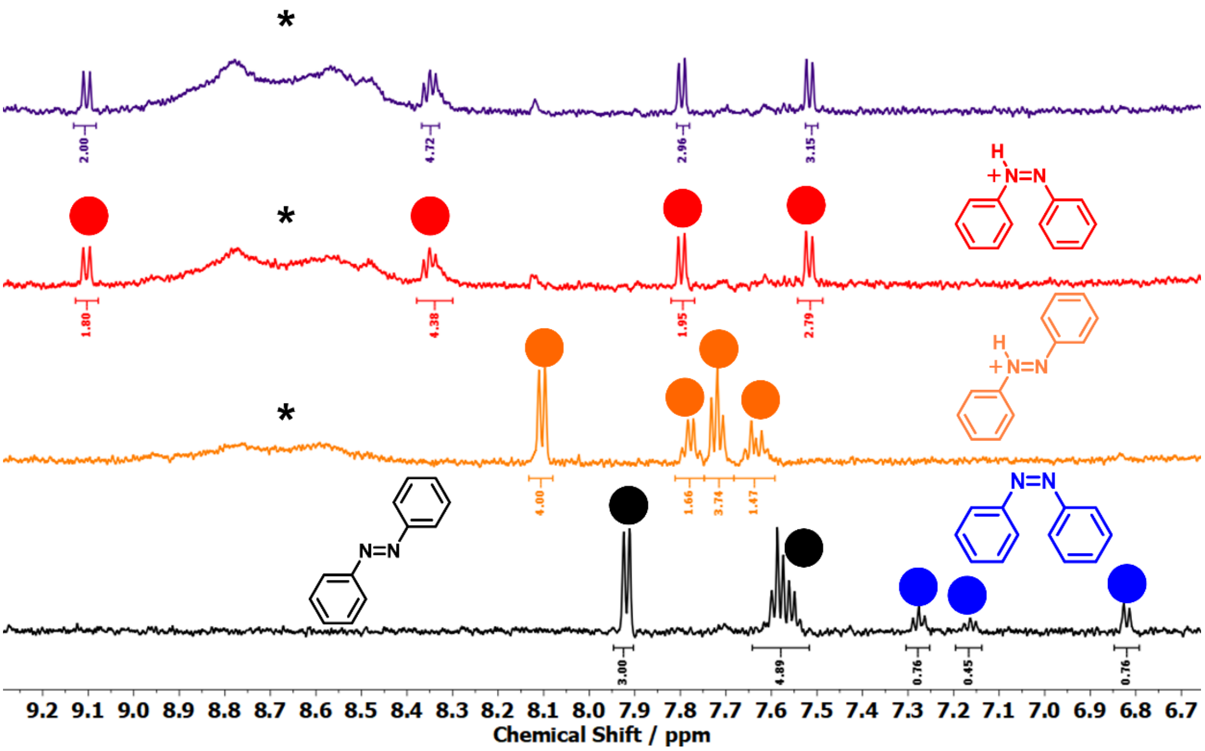

Figure S14. ^{1}H-NMR spectra of AB under ambient conditions (black, 25 µM, 600 MHz, ACN-d_3, δ 7.94 – 7.89 (m, 4H), 7.62 – 7.51 (m, 6H), 7.31 – 7.24 (m, 0H), 7.16 (t, J = 7.2 Hz, 0H), 6.82 (dd, J = 8.5, 1.3 Hz, 0H)), after addition of 500 equivalents of TfOH (orange, 25 µM, 600 MHz, ACN-d_3, δ 8.10 (d, J = 8.0 Hz, 4H), 7.78 (d, J = 7.1 Hz, 2H), 7.72 (t, J = 7.6 Hz, 2H), 7.66 – 7.61 (m, 2H)), irradiation with UV light (red, 25 µM, 600 MHz, ACN-d_3, δ 9.12 – 9.09 (d, 2H), δ 8.38 – 8.32 (m, 4H), 7.80 (d, J = 8.1 Hz, 2H), 7.52 (d, J = 8.2 Hz, 2H)) and irradiation with green light (purple, 25 µM, 600 MHz, ACN-d_3, δ 9.12 – 9.09 (d, 2H), δ 8.38 – 8.32 (m, 4H), 7.80 (d, J = 8.1 Hz, 2H), 7.52 (d, J = 8.0 Hz, 2H)). We note that in the case of ABH^+, unlike with MeO-ABH^+, the photoswitching thus seems to be one-way to the *Z*-ABH^+ species. * broad peaks from interaction with 500 eq. TfOH.

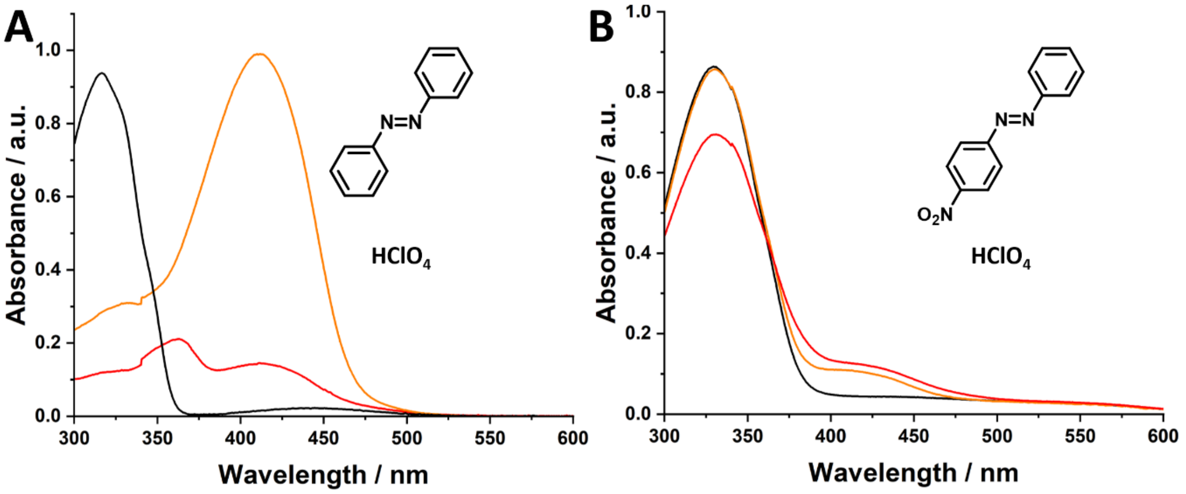

Figure S15. UV-vis spectra of AB (A) and *p*-O_2N-AB (B) at 25 µM in acetonitrile; Measurements were performed in the following order: ambient conditions (black), after addition of 500 equivalents of $HClO_4$ (orange) and after irradiation with 365 nm (red).

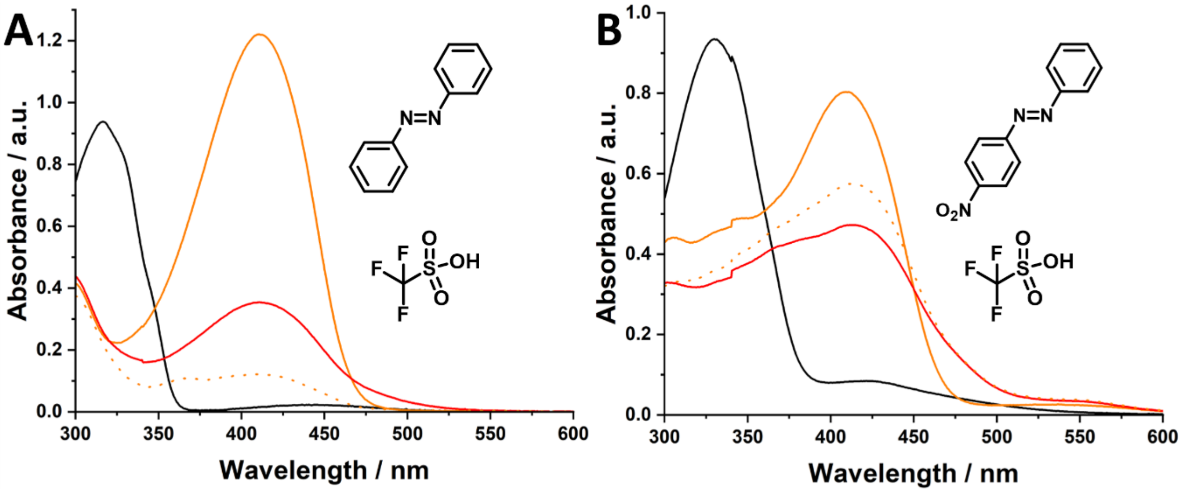

Figure S16. UV-vis spectra of AB (A) and *p*-O_2N-AB (B) at 25 µM in acetonitrile; Measurements were performed in the following order: ambient conditions (black), after addition of 500 equivalents of TfOH (orange), after irradiation with 365 nm (red) and after irradiation with 520 nm (orange, dotted).

Table S2. ^{1}H-NMR chemical shifts (in ppm) and corresponding coupling constants (in Hz) of the protonated and unprotonated isomers of MeO-AB (0.2 mM in ACN-d_3, spectra in Figure 3B and D).

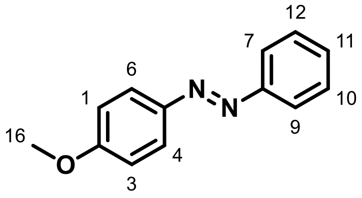	Ar-$H_{7/9}$	Ar-$H_{4/6}$	Ar-$H_{10/11/12}$	Ar-$H_{1/3}$	-OCH_{16}
E	7.91 (d) 9.33 Hz	7.86 (d) 7.12 Hz	7.54 (m)	7.10 (d) 9.38 Hz	3.89 (s)
Z	7.31 (t) 7.87 Hz	7.18 (t) 7.42 Hz	6.83 (m)		3.74 (s)
EH^+	7.92 (d) J = 9.18 Hz	7.69 (m)	8.02 (dd) J = 2.52, 3.70, 3.65 Hz	8.15 (d) J = 9.18 Hz	4.01 (s)
ZH^+	7.38 (d) J = 9.31 Hz	7.75 (m)	8.12 (d) 1.54 Hz	8.31 (d) J = 9.28 Hz	4.10 (s)

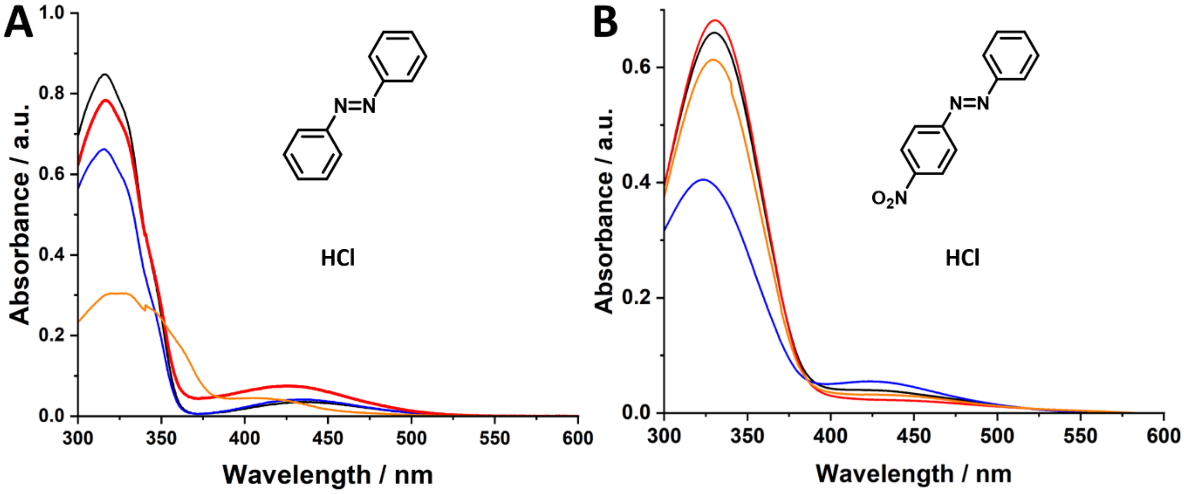

Figure S17. UV-vis spectra of AB (A) and *p*-O_2N-AB (B) at 25 µM in acetonitrile; Measurements were performed in the following order: ambient conditions (black), irradiation with 365 nm (blue), addition of 500 equivalents of HCl (red), another irradiation with 365 nm (orange).

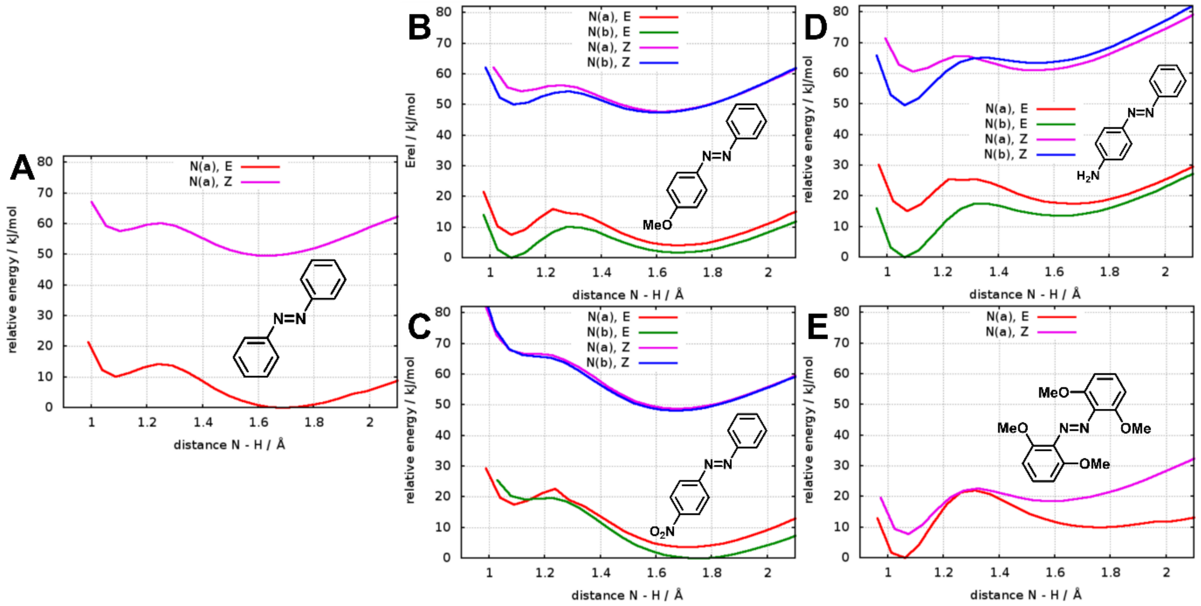

Figure S18. Relative energies in kJ*mol^{-1} vs Proton-Azo-N distance in Å of given azobenzene species. Here N(a) is bearing the phenyl ring including the substituent(s) and N(b) bears an unsubstituted phenyl ring. (A) AB, (B) MeO-AB, (C) O_2N-AB, (D) H_2N-AB, (E) tetra-MeO-AB.

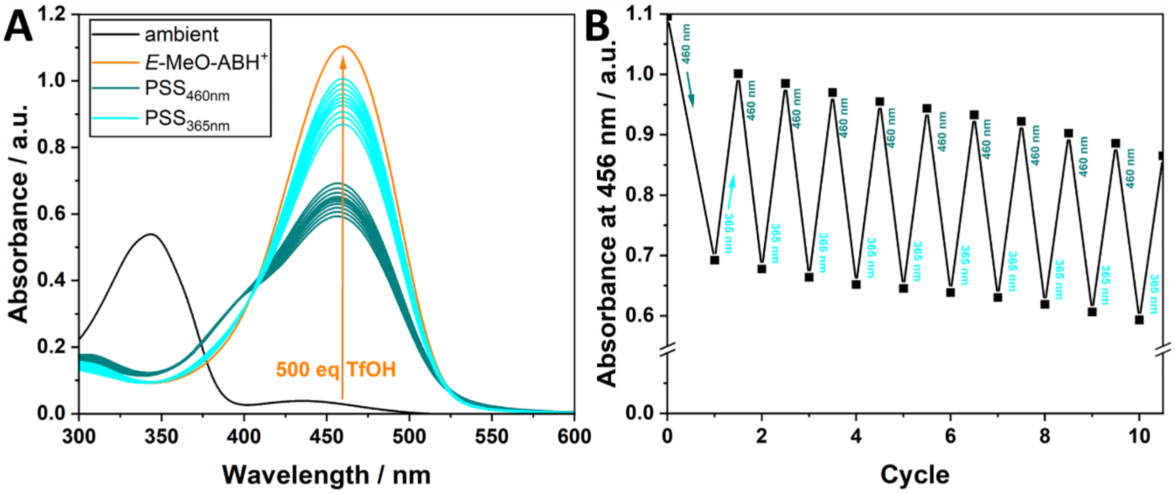

Figure S19. (A) UV-vis spectra of MeO-AB as ambient (black) and after addition of excess TfOH (orange), at PSS_{365nm} (bright blue) and at PSS_{460nm} (turquoise) (at 25 µM in acetonitrile) and (B) absorbance at λ_{max}456 nm plotted against ten cycles of alternating irradiation.

Table S3. Cartesian coordinates for *E* MeO-AB.

E(RB3LYP) = -687.347591472 ; Sum of electronic and thermal Free Energies= -687.165899; NImag = 0

C	-0.84181000	0.14820100	-0.00004600
N	0.52782300	0.47188400	-0.00000900
N	1.31607700	-0.51689100	0.00004800
C	2.69174700	-0.17841000	0.00002300
C	-1.36464700	-1.16205500	-0.00052900
C	-1.72548900	1.23411500	0.00037800
C	-2.73088500	-1.36258900	-0.00055100
C	-3.10489700	1.04300000	0.00039700
C	-3.61424300	-0.26282700	-0.00009300
H	-1.31007100	2.23702000	0.00072700
H	-3.76463600	1.90139700	0.00078500
O	-4.93321800	-0.57426600	-0.00011400
C	3.19503900	1.13445700	-0.00050000
C	3.57864300	-1.26372800	0.00052200
C	4.56899300	1.34484100	-0.00049000
C	4.95559200	-1.04627700	0.00054200
C	5.45361100	0.25809300	0.00003700
H	-0.67952000	-2.00181800	-0.00088700
H	-3.15288000	-2.36257000	-0.00095100
H	2.49798200	1.96442800	-0.00091000
H	3.16547000	-2.26769800	0.00089700
H	4.95855800	2.35889100	-0.00090400
H	5.63742000	-1.89141500	0.00094100
H	6.52567000	0.43159800	0.00003300
C	-5.88037900	0.49411700	0.00027700
H	-6.86191100	0.01987200	0.00010300
H	-5.77267000	1.11659200	0.89562600
H	-5.77261800	1.11725600	-0.89461000

Table S4. Cartesian coordinates for *Z* MeO-AB.

E(RB3LYP) = -687.329226279; Sum of electronic and thermal Free Energies= -687.146836; NImag = 0

C	0.44444500	1.27374900	-0.10757900
N	-0.59752000	2.24583600	-0.02186900
N	-1.81976200	1.96138700	-0.03497400
C	-2.31281700	0.61911700	0.07163400
C	0.42426200	0.14893300	-0.95471600
C	1.61415800	1.57260300	0.59849000
C	1.53878800	-0.66567500	-1.05066300
C	2.72466700	0.73360100	0.54158300

C	2.69047900	-0.39458500	-0.28918400
H	1.63789600	2.46667900	1.21378900
H	3.60581700	0.97526700	1.12258000
O	3.71578100	-1.27050400	-0.44184300
C	-1.93133000	-0.22668400	1.12325900
C	-3.32531300	0.22928100	-0.81186800
C	-2.54151300	-1.47076600	1.26043100
C	-3.90683100	-1.03193000	-0.68657900
C	-3.51869400	-1.88441000	0.34974700
H	-0.45328900	-0.07260500	-1.54975500
H	1.54444400	-1.52625900	-1.71163400
H	-1.16802500	0.09405200	1.82411100
H	-3.63298700	0.91214400	-1.59790300
H	-2.25028100	-2.12206100	2.07934700
H	-4.67387600	-1.34215200	-1.38997700
H	-3.98372100	-2.85974400	0.45615200
C	4.91428000	-1.03960100	0.29774000
H	5.58806400	-1.85468800	0.03283200
H	5.37045000	-0.08184800	0.02255800
H	4.72274700	-1.05710400	1.37676100

Table S5. Cartesian coordinates for *E* MeO-AB-H(a)$^{+}$.

E(RB3LYP) = -687.786219207; Sum of electronic and thermal Free Energies= -687.590344; NImag = 0

C	0.89073000	-0.14564400	-0.00307000
N	-0.47928400	-0.40448300	-0.00696500
N	-1.36594800	0.50174000	0.00130200
C	-2.70558500	0.15991700	0.00278400
C	1.38606700	1.17294700	0.01774800
C	1.76144000	-1.24499800	-0.02005900
C	2.74734900	1.37291600	0.02074900
C	3.13193000	-1.04177800	-0.01689500
C	3.63601400	0.27163000	0.00358900
H	1.36765800	-2.25685800	-0.03559000
H	3.79549800	-1.89583600	-0.03015700
O	4.93896000	0.58474300	0.00873200
C	-3.23624600	-1.15112700	0.02486600
C	-3.57176100	1.27355600	-0.01829500
C	-4.61028200	-1.32296100	0.02173900
C	-4.94741800	1.08534400	-0.02253700
C	-5.46714900	-0.21105500	-0.00283400
H	0.69712100	2.00825900	0.03122700
H	3.16546500	2.37291100	0.03669700
H	-2.60189300	-2.03152100	0.04921900
H	-3.13301200	2.26493000	-0.03283100

H	-5.02495900	-2.32493200	0.03995900
H	-5.61205400	1.94208700	-0.04019400
H	-6.54152300	-0.36350800	-0.00481000
C	5.90874000	-0.47214400	-0.00679300
H	6.87834500	0.02367000	0.00007800
H	5.80723500	-1.10367200	0.88118200
H	5.80674500	-1.07777500	-0.91257200
H	-0.74265300	-1.39612600	-0.01838700

Table S6. Cartesian coordinates for *E* MeO-AB-H(b)$^+$.

E(RB3LYP) = -687.790597074; Sum of electronic and thermal Free Energies= -687.592749; NImag = 0

C	-0.86610900	0.09777300	0.00015700
N	0.45283700	0.42384800	0.00011700
N	1.35418800	-0.47627600	-0.00017500
C	2.73090600	-0.17623900	-0.00011400
C	-1.42240400	-1.21545600	0.00031100
C	-1.73619000	1.21777100	0.00007300
C	-2.78243800	-1.37507900	0.00026600
C	-3.10565900	1.06110200	-0.00001700
C	-3.64296800	-0.24297400	0.00005200
H	-1.29065200	2.20618900	0.00001100
H	-3.74911000	1.93059100	-0.00012900
O	-4.94256400	-0.52349500	-0.00003400
C	3.17991200	1.15059000	0.00006000
C	3.62411300	-1.25414300	-0.00022100
C	4.54862200	1.38793800	0.00012400
C	4.99188300	-0.99596200	-0.00015100
C	5.45606800	0.32110900	0.00002300
H	-0.79842700	-2.10304400	0.00056500
H	-3.23427600	-2.36026400	0.00041500
H	2.46449100	1.96346800	0.00013800
H	3.25431900	-2.27527300	-0.00034400
H	4.91203900	2.41021700	0.00025100
H	5.69234200	-1.82419200	-0.00022800
H	6.52296300	0.51909700	0.00008000
C	-5.90012200	0.55094500	-0.00030700
H	-6.87466100	0.06616800	-0.00052200
H	-5.78562900	1.16415800	0.89764500
H	-5.78521800	1.16409500	-0.89824600
H	1.10946300	-1.47051400	-0.00050600

Table S7. Cartesian coordinates for *Z* MeO-AB-H(a)$^+$.

E(RB3LYP) = -687.772287992; Sum of electronic and thermal Free Energies= -687.573818; NImag = 0

C	0.59219600	1.07694800	-0.04546700
N	-0.51846000	1.94771700	0.01474000
N	-1.77669400	1.79590700	-0.07045200
C	-2.41794400	0.56924200	0.03311500
C	0.57687700	-0.13396000	-0.76542700
C	1.77727100	1.54200200	0.54263300
C	1.73491500	-0.87225200	-0.85709900
C	2.93778900	0.78767700	0.46961700
C	2.92392700	-0.43107800	-0.23117900
H	1.78421200	2.48933900	1.07319800
H	3.83907600	1.15216600	0.94402600
O	3.98364400	-1.23729300	-0.37640300
C	-1.97503600	-0.52970600	0.80488800
C	-3.68997000	0.53967000	-0.57583600
C	-2.79383000	-1.64034400	0.92673100
C	-4.47456300	-0.60326200	-0.48912200
C	-4.02920100	-1.69248700	0.26353700
H	-0.31879000	-0.47541100	-1.26697200
H	1.75907500	-1.79873200	-1.41914400
H	-1.03001300	-0.49470300	1.33142200
H	-4.02356500	1.41532600	-1.12138700
H	-2.47167200	-2.47615100	1.53857200
H	-5.43876100	-0.63679300	-0.98448800
H	-4.64896500	-2.57890900	0.35187600
C	5.23167100	-0.86002800	0.22245600
H	5.92617800	-1.66123300	-0.02588300
H	5.59043200	0.08604300	-0.19458000
H	5.13041800	-0.77836100	1.30901500
H	-0.26531800	2.93585400	0.07246900

Table S8. Cartesian coordinates for *Z* MeO-AB-H(b)$^+$.

E(RB3LYP) = -687.778008070; Sum of electronic and thermal Free Energies= -687.579978; NImag = 0

C	-0.56853200	-1.11213200	-0.00401800
N	0.39555300	-2.06381200	0.07116900
N	1.65469100	-1.85649000	0.01760100
C	2.41138800	-0.63084200	0.05084600
C	-0.45093100	0.27440400	-0.34138300
C	-1.86698200	-1.64903800	0.22824700
C	-1.57617000	1.04997500	-0.41622700
C	-2.99618600	-0.86341900	0.18274100
C	-2.85920200	0.50368300	-0.13957000

H	-1.93935200	-2.70644000	0.45593500
H	-3.96709100	-1.29826500	0.37885000
O	-3.86823700	1.36178800	-0.22257500
C	2.33750200	0.18442100	1.18322000
C	3.23931700	-0.32490800	-1.02820300
C	3.09598800	1.35129500	1.21455300
C	3.99162100	0.84933600	-0.98175800
C	3.91706300	1.68613300	0.13310400
H	0.50615700	0.71714800	-0.57022000
H	-1.51630000	2.09778400	-0.68731600
H	1.69917700	-0.09205400	2.01535800
H	3.27975500	-0.98351900	-1.88930800
H	3.05085000	1.99530600	2.08650300
H	4.63293800	1.10733700	-1.81792600
H	4.50422100	2.59828500	0.16400400
C	-5.21095900	0.91014500	0.03793800
H	-5.83742900	1.79178000	-0.08354900
H	-5.50025000	0.14104900	-0.68309300
H	-5.29267900	0.52795300	1.05883600
H	2.19068300	-2.72285900	0.01420300

SUPPORTING EXPERIMENTAL PROCEDURES

General procedures

All chemicals and solvents were purchased from Sigma-Aldrich (Sigma-Aldrich Corp., St. Louis, Missouri, USA), Acros Organics (Fisher Scientific International, Inc., Pittsburgh, Pennsylvania, USA) and TCI (Tokyo Chemical Industry, Tokyo, Japan) and were used without further purification.

UV-vis spectroscopy

UV/vis spectra were measured on a Jasco V-770 spectrophotometer (Jasco Deutschland GmbH, Pfungstadt, Germany) using High Precision quartz glass cuvettes (Hellma Analytics GmbH, Müllheim, Germany). The spectra were recorded with Spectra Manager 2, Spectra Manager Version 2.14.06 (Jasco Deutschland GmbH, Pfungstadt, Germany). The samples were dissolved in the specified solvent and the baseline was measured against the same solvent. Data analysis was done using OriginPro 2018 b b9.5.5.409 (ORIGINLAB Corperation, Northampton, USA). If not stated otherwise, the *Z*-isomer was handled in the dark or under red light.

Irradiation experiments were conducted using LEDs with emission wavelengths of 365 nm (UV LED Gen2 Emitter, LED Engin Inc., San Jose, California, USA, radiant flux 1.2 W), 460 nm (Blue LED Emitter, LED Engin Inc., San Jose, California, USA, radiant flux 1.0 W) and 520 nm (LSC-G HighPower-LED, Cree Inc., Durham, North Carolina, USA, radiant flux 87 lm) at room temperature. Irradiation times of 10 s (MeO-AB) and 20 s (AB and NO_2-AB) and a distance between light source and sample of 1 cm were used to isomerize the compounds directly inside the UV-cuvettes.

^{1}H-NMR spectroscopy

The NMR spectra were obtained using a DD2-600-spectrometer with 600 Hz (Agilent Technologies, Santa Clara, California, USA). Chemical shifts (δ) are reported in parts per million with respect to tetramethylsilane, referenced to residual solvent (CD_2HCN) signals, and coupling constants are denoted in hertz. Integrations are reported, with multiplicities denoted as: s = singlet, d = doublet, t = triplet, br = broad singlet, m = multiplet. MestReNova 14.2.0-26256 (Mestrelab Research S.L., Santiago de Compostela, Spain) was used to analyze all NMR spectra.

DFT calculations

All DFT calculations were performed with the Gaussian 09 Rev. D.01 package[3] employing the B3LYP hybrid functional[4] and the 6-31G* basis set.[5] The standard convergence criteria for geometry optimizations and single point calculations were used, along with the addition of empirical dispersion correction of type Grimme D3 with Becke-Johnson damping.[6–8] No counterions were employed for the cationic species. To include solvent effects of Acetonitrile into the calculations, the polarizable continuum model (PCM)[9–11] was used and the cavity for the molecule was formed based on the UFF model for atomic radii.[12] UV/vis spectra were obtained for the ground state optimized structures from TD-DFT calculations of the lowest 100 excited states employing a Lorentzian line broadening of 50 nm (fwhm). For the visualization of molecular geometries VMD 1.9.3 with the internal Tachyon renderer was used.[13,14] Minimum energy profiles for the ground state along the dihedral angle coordinate CNNC in the interval [70,110] degrees were calculated starting from the *E*- and *Z*-

optimized structures, respectively, followed by a harmonic fit to yield the transition state energy. Gibbs free energies were obtained accordingly by including zero point and thermodynamic corrections resulting from additional harmonic frequency calculations for standard temperature T= 298.15 K.

Supporting References

(1) Dong, M.; Babalhavaeji, A.; Samanta, S.; Beharry, A. A.; Woolley, G. A. Red-Shifting Azobenzene Photoswitches for in Vivo Use. *Accounts of Chemical Research* **2015**, *48*, 2662–2670.

(2) Bléger, D.; Schwarz, J.; Brouwer, A. M.; Hecht, S. o-Fluoroazobenzenes as Readily Synthesized Photoswitches Offering Nearly Quantitative Two-Way Isomerization with Visible Light. *Journal of the American Chemical Society* **2012**, *134*, 20597–20600.

(3) Frisch, M. J.; Trucks, G. W.; Schlegel, H. B.; Scuseria, G. E.; Robb, M. A.; Cheeseman, J. R.; Scalmani, G.; Barone, V.; Petersson, G. A.; Nakatsuji, H.*; et al. Gaussian 09 Rev. D.01*; Wallingford, CT, 2013.

(4) Stephens, P. J.; Devlin, F. J.; Chabalowski, C. F.; Frisch, M. J. Ab Initio Calculation of Vibrational Absorption and Circular Dichroism Spectra Using Density Functional Force Fields. *The Journal of Physical Chemistry* **1994**, *98*, 11623–11627.

(5) Francl, M. M.; Pietro, W. J.; Hehre, W. J.; Binkley, J. S.; Gordon, M. S.; DeFrees, D. J.; Pople, J. A. Self-consistent molecular orbital methods. XXIII. A polarization-type basis set for second-row elements. *The Journal of Chemical Physics* **1982**, *77*, 3654–3665.

(6) Grimme, S.; Antony, J.; Ehrlich, S.; Krieg, H. A consistent and accurate ab initio parametrization of density functional dispersion correction (DFT-D) for the 94 elements H-Pu. *The Journal of Chemical Physics* **2010**, *132*, 154104.

(7) Grimme, S.; Ehrlich, S.; Goerigk, L. Effect of the damping function in dispersion corrected density functional theory. *J. Comput. Chem.* **2011**, *32*, 1456–1465.

(8) Smith, D. G. A.; Burns, L. A.; Patkowski, K.; Sherrill, C. D. Revised Damping Parameters for the D3 Dispersion Correction to Density Functional Theory. *The Journal of Physical Chemistry Letters* **2016**, *7*, 2197–2203.

(9) Miertuš, S.; Scrocco, E.; Tomasi, J. Electrostatic interaction of a solute with a continuum. A direct utilizaion of AB initio molecular potentials for the prevision of solvent effects. *Chemical Physics* **1981**, *55*, 117–129.

(10) Improta, R.; Barone, V.; Scalmani, G.; Frisch, M. J. A state-specific polarizable continuum model time dependent density functional theory method for excited state calculations in solution. *The Journal of Chemical Physics* **2006**, *125*, 54103.

(11) Improta, R.; Scalmani, G.; Frisch, M. J.; Barone, V. Toward effective and reliable fluorescence energies in solution by a new state specific polarizable continuum model time dependent density functional theory approach. *The Journal of Chemical Physics* **2007**, *127*, 74504.

(12) Rappe, A. K.; Casewit, C. J.; Colwell, K. S.; Goddard, W. A.; Skiff, W. M. UFF, a full periodic table force field for molecular mechanics and molecular dynamics simulations. *Journal of the American Chemical Society* **1992**, *114*, 10024–10035.

(13) Humphrey, W.; Dalke, A.; Schulten, K. VMD: visual molecular dynamics. *Journal of molecular graphics* **1996**, *14*, 33-8, 27-8.

(14) Stone, J. An Efficient Library for Parallel Ray Tracing and Animation **1997**.
